# *Tripterygium wilfordii* Hook. f. and Xinfeng capsule in rheumatoid arthritis: a review of clinical evidence, molecular mechanisms, and translational perspectives

**DOI:** 10.3389/fimmu.2026.1814416

**Published:** 2026-06-18

**Authors:** Jianting Wen, Jian Liu

**Affiliations:** 1 Department of Rheumatology and Immunology, First Affiliated Hospital of Anhui University of Chinese Medicine, Hefei, Anhui, China; 2 Institute of Rheumatology, Anhui Academy of Chinese Medicine, Hefei, Anhui, China; 3 Anhui Provincial Key Laboratory of Basic Research and Clinical Translation in Rheumatology of Traditional Chinese Medicine, Hefei, Anhui, China

**Keywords:** clinical efficacy, molecular mechanisms, rheumatoid arthritis, *Tripterygium wilfordii* Hook. f., Xinfeng capsule

## Abstract

*Tripterygium wilfordii* Hook. f. (TWHf) has long been used in traditional medicine to treat rheumatoid arthritis (RA); however, but its clinical application needsrequires a balanced understanding of its efficacy, mechanismsms, and safety. This review aimed to summarize the efficacy, mechanisms, clinical evidence, and translational challenges of TWHf-derived monomers, Tripterygium Glycosides Tablets (TGT), and Xinfeng Capsule (XFC) in RA. We searched relevant studies in PubMed, Web of Science, Scopus, Embase, and CNKI from database inception to [insert search date] for eligible studies. These include clinical trials, preclinical studies, pharmacological investigations, and mechanistic studies on TWHf/XFC in RA. Preclinical evidence indicates that TWHf-derived compounds regulate multiple RA-related processes, including the NF-κB and PI3K/AKT/mTOR signaling pathways, ROS-NLRP3 inflammasome activation, synovial hyperplasia, angiogenesis, and immune cell dysfunction. Compared with isolated monomers, XFC exhibits broader multi-component and multi-target regulation, involving epigenetic modulation, NETosis, cuproptosis, pyroptosis, oxidative stress, and coagulation homeostasis, which may enhance efficacy and reduce toxicity via compound compatibility. Clinical studies suggest that TWHf-related preparations may improve disease activity, joint symptoms, and inflammatory markers, whether used alone or in combination with conventional DMARDs or biologics. However, several major challenges remain, such as toxicity, insufficient high-quality multicenter RCTs, limited standardization, and a lack of personalized dosing. Future studies should focus on standardized formulations, rigorous clinical evaluation, safety monitoring, and toxicity-attenuating delivery systems, as well as biomarker-guided precision application of TWHf and XFC in RA.

## Graphical Abstract

**Figure:**
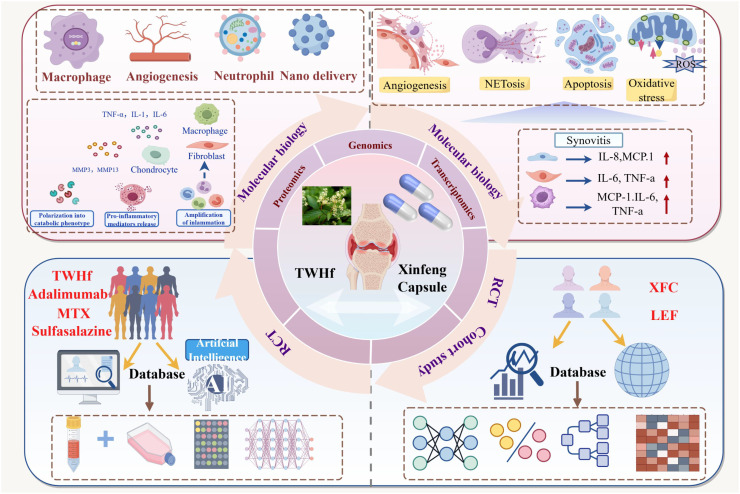
Therapeutic landscape of RA and positioning of TWHf-based therapies

## 1 Introduction

Rheumatoid arthritis (RA) is a chronic, progressive autoimmune disease characterized by persistent synovial inflammation, destructive proliferation of synovial fibroblasts, and gradual erosion of articular cartilage and bone. These pathological changes ultimately lead to irreversible joint deformity, functional disability, and severe impairment of health-related quality of life (1–4). The global prevalence of RA ranges from 0.5% to 1%, with a female predominance (female-to-male ratio: approximately 3:1). RA places a substantial socioeconomic burden on individuals, families, and healthcare systems worldwide, encompassing long-term medical expenditures, lost productivity, and reduced life expectancy (5, 6). The pathological landscape of RA is highly complex and not yet fully elucidated. It involves three key abnormalities: (1) dysregulated interactions among innate and adaptive immune cells (e.g., T lymphocytes, B lymphocytes, macrophages, and neutrophils); (2) excessive production of pro-inflammatory cytokines [such as tumor necrosis factor (TNF)-α, interleukin (IL)-6, and IL-1]; and (3) aberrant activation of signaling pathways that drive synovial hyperplasia and tissue destruction (7–10). Despite advances in understanding RA pathogenesis, the precise molecular triggers that initiate the autoimmune response remain unclear—a gap that hinders the development of curative therapies. Current clinical management of RA follows a hierarchical approach involving non-steroidal anti-inflammatory drugs (NSAIDs), conventional synthetic disease-modifying antirheumatic drugs (csDMARDs), and biological DMARDs (bDMARDs) (11). NSAIDs provide rapid symptomatic relief by inhibiting cyclooxygenase enzymes and reducing inflammatory mediator release (12). However, they do not alter disease progression and are associated with gastrointestinal, cardiovascular, and renal adverse effects with long-term use. csDMARDs, represented by methotrexate (MTX), remain the first-line disease-modifying therapy for RA. They exert immunomodulatory effects to slow joint damage. However, their clinical efficacy is limited by three major drawbacks: (1) delayed onset of action (3-6 months); (2) inadequate response in approximately 30-40% of patients; and (3) potential toxicities including hepatotoxicity, myelosuppression, and teratogenicity (13). DMARDs (including anti-TNF agents, anti-IL-6 receptor antibodies, and B-cell-depleting therapies) offer targeted efficacy for refractory RA by blocking key pro-inflammatory pathways. However, their widespread use is constrained by multiple factors, such as high cost, increased risk of infections (particularly opportunistic pathogens), loss of response over time, and contraindications in patients with comorbidities (e.g., malignancy or chronic infections) (14). Collectively, these therapeutic limitations underscore the urgent need for novel agents with improved efficacy, favorable safety, and mechanisms that address the multifactorial pathogenesis of RA.

*Tripterygium wilfordii* Hook. f. (TWHf), a traditional Chinese medicine (TCM) belonging to the Celastraceae family. It has a long history of use in TCM for treating inflammatory and autoimmune disorders, first documented in the Ming Dynasty classic *Ben Cao Gang Mu* (Compendium of Materia Medica), where it was recorded for alleviating joint swelling, pain, and stiffness. In TCM theory, TWHf has a “bitter taste and cold nature” and acts on the liver and kidney meridians. It exerts therapeutic effects of “dispelling wind-dampness, dredging collaterals, and relieving pain”. These effects align with the pathological manifestations of RA, which is categorized as “bi syndrome” (arthralgia syndrome) in TCM. With the advancement of modern pharmacology, extensive studies have revealed that TWHf has abundant bioactive secondary metabolites, predominantly classified into diterpenoids, triterpenoids, and alkaloids. These compounds are responsible for its diverse pharmacological activities, including potent anti-inflammatory, immunomodulatory, anti-proliferative, and analgesic effects (15, 16). Particularly in RA, accumulating evidence has demonstrated that TWHf-derived monomers exert therapeutic effects by targeting multiple pathological processes of the disease. These include: (1) inhibiting the activation and proliferation of synovial fibroblasts; (2) suppressing the production of pro-inflammatory cytokines (e.g., TNF-α, IL-6, and IL-1β); (3) regulating the balance of immune cell subsets (e.g., T cells, B cells, and macrophages); and (4) blocking aberrant signaling pathways involved in inflammation and tissue destruction (e.g., NF-κB, MAPK, JAK/STAT pathways) (17, 18).

As a representative compound preparation of TWHf, Xinfeng Capsule (XFC) is a hospital-prepared TCM formulation developed by the First Affiliated Hospital of Anhui University of Chinese Medicine (Approval Number: ZL201310011369.8, Wan Yao Zhi Zi: Z20050062). It has a clinical application history of over three decades in RA treatment (19). XFC is developed based on the TCM principle of “treating the root cause and relieving symptoms simultaneously”. It consists of four medicinal herbs: Astragali Radix et Rhizoma (Huangqi), Coicis Semen (Yiyiren), Tripterygii Radix et Rhizoma (Leigongteng), and Scolopendra (Wugong). These herbs synergistically exert the effects of “invigorating spleen qi, resolving dampness, dredging collaterals, and relieving arthralgia.” This rational compatibility is designed to address the core pathogenesis of RA in TCM: “spleen deficiency with dampness stagnation, and collateral obstruction by blood stasis”. In this formulation, Astragali Radix et Rhizoma tonifies spleen qi to strengthen the body’s resistance; Coicis Semen invigorates the spleen and eliminates dampness; Tripterygii Radix et Rhizoma dispels wind-dampness and dredges collaterals; and Scolopendra penetrates collaterals to relieve pain (20). Clinical practice over the past three decades has consistently confirmed the remarkable efficacy of XFC in RA patients. It improves joint symptoms, reduces inflammatory markers, and delays disease progression, while demonstrating a more favorable safety profile compared to single TWHf monomers. These characteristics make XFC a typical example of TCM compound formulations that leverage the “multi-component, multi-target, and synergistic regulation” advantage. Thus, XFC provides a valuable therapeutic option for RA and lays a solid foundation for further mechanistic exploration and clinical translation (21, 22).

Therefore, a focused synthesis of TWHf-derived monomers, standardized preparations, and XFC is needed to clarify their pharmacological basis, clinical evidence, safety concerns, and translational limitations in RA. Unlike previous summaries that mainly described individual compounds or isolated signaling pathways, this review emphasized both monomer-based mechanisms and the compound compatibility features of XFC. Specifically, we summarized the chemical classification and pharmacological activities of representative TWHf-derived agents and evaluated their clinical and preclinical evidence. Additionally, we discussed safety and standardization challenges and highlighted future directions for evidence-based and precision-oriented applications in RA.

This schematic summarizes the clinical evidence, pharmacological mechanisms, and translational strategies of TWHf-derived preparations and XFC in RA. The left part illustrates TWHf-related interventions, including comparisons with adalimumab, MTX, and sulfasalazine in clinical studies, as well as mechanistic actions involving macrophage regulation, angiogenesis, neutrophil activity, chondrocyte protection, FLS modulation, and nanodelivery-based optimization. The right part highlights XFC-related evidence from RCT and cohort studies, including comparisons with LEF, and summarizes its integrated mechanisms involving angiogenesis, NETosis, apoptosis, oxidative stress, inflammatory cytokine regulation, and synovitis improvement. The lower panels indicate the use of literature databases, data mining, and AI-assisted approaches for evidence integration and mechanistic prediction. This figure was generated based on studies summarized in this review and does not contain original quantitative data; therefore, no statistical analysis was performed. Abbreviations: RA, rheumatoid arthritis; TWHf, *Tripterygium wilfordii* Hook. f.; XFC, Xinfeng Capsule; MTX, methotrexate; LEF, leflunomide; RCT, randomized controlled trial; NETosis, neutrophil extracellular trap formation; ROS, reactive oxygen species; IL, interleukin; TNF-α, tumor necrosis factor-α; MCP-1, monocyte chemoattractant protein-1; AI, artificial intelligence.

## 2 Literature search strategy and evidence assessment

A narrative literature review was conducted to summarize the clinical evidence, molecular mechanisms, safety profile, and translational potential of TWHf. TWHf-derived monomers, Tripterygium Glycosides Tablets (TGT), and XFC in RA. Relevant studies were searched in PubMed, Web of Science, Scopus, Embase, and CNKI from database inception to [March 31, 2026], using the search terms of “Tripterygium wilfordii, triptolide, celastrol, Tripterygium glycosides, Xinfeng Capsule, and rheumatoid arthritis. Studies were included if they were directly related to RA efficacy, safety, or molecular mechanisms. These included clinical studies, randomized controlled trials (RCTs), animal and cellular experiments, network pharmacology analyses, omics-based studies, and pharmacological investigations. Studies were excluded if they were unrelated to RA, lacked sufficient methodological information, or did not involve TWHf-related preparations or XFC. The included literature was narratively synthesized according to intervention type, evidence category, mechanism, safety, and translational relevance.

## 3 Chemical classification and physicochemical properties of core TWHf-derived monomers

**Figure 1:**
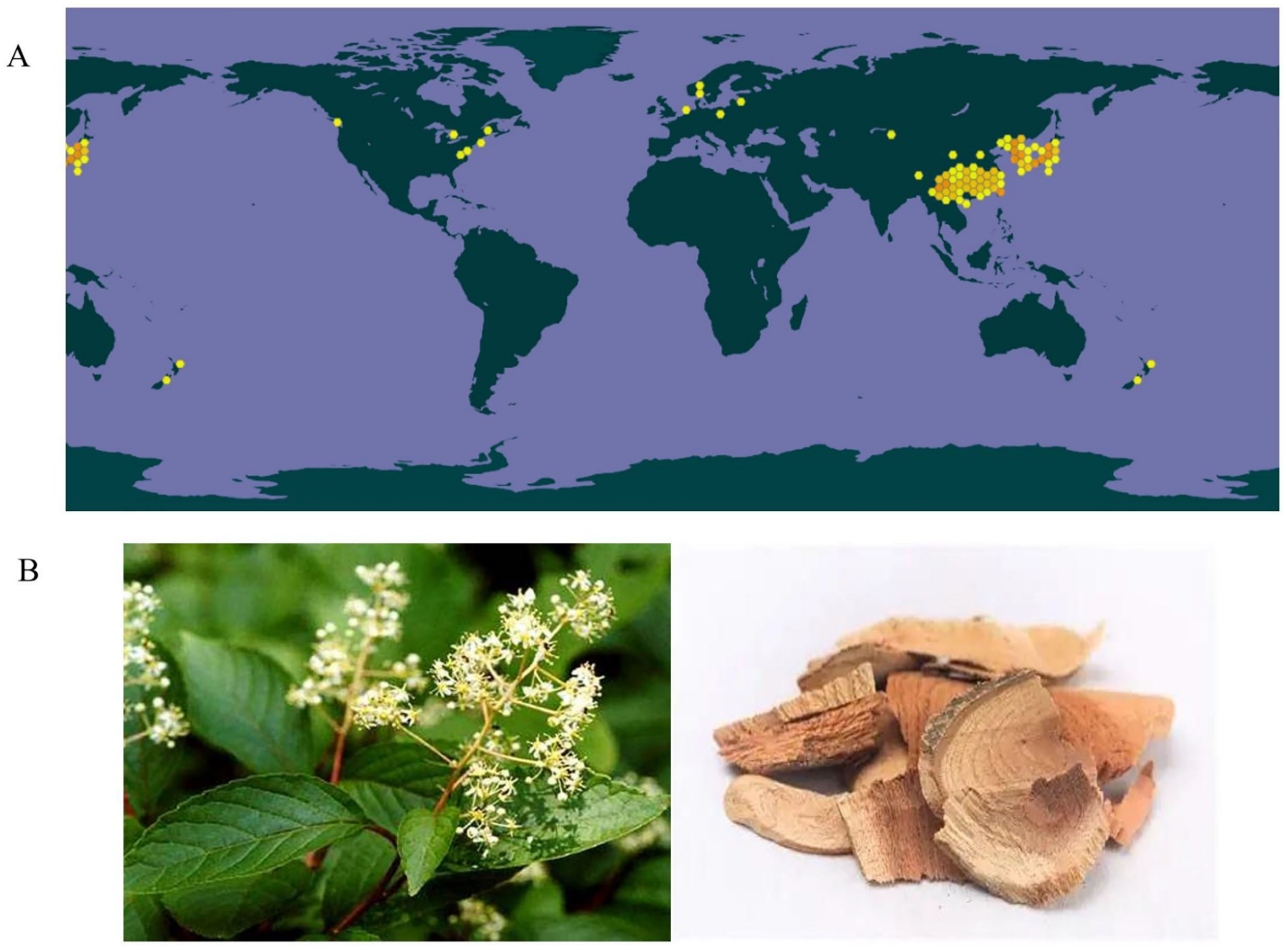
Geographical distribution of TWHf worldwide. **(A)** Global geographical distribution of TWHf. The yellow dots indicate reported distribution or occurrence sites, with a predominant concentration in East and Southeast Asia. **(B)** Representative images of TWHf, showing the whole plant with inflorescences and the dried medicinal root material commonly used in TCM preparations. This figure is a schematic summary based on publicly available distribution information and representative morphological images. No original quantitative data or statistical analysis is included.

The diverse pharmacological activities of TWHf are mainly attributed to its abundant bioactive secondary metabolites. These are structurally categorized into three major classes: diterpenoids, triterpenoids, and alkaloids. These compounds have distinct chemical skeletons and physicochemical profiles, which lay the foundation for their specific biological effects and pharmaceutical development (summarized in **Table 1**; **Figure 1**).

**Table 1:** Chemical classification and structural diversity of bioactive monomers from TWHf.

Compound name	Chemical formula	Molecular weight (Da)	Key physicochemical properties	Chemical structure (skeleton)
TPL	C_20_H_24_O_6_	360.4	Abietane-type diterpenoid with an epoxy-containing skeleton; poorly water-soluble but highly bioactive.	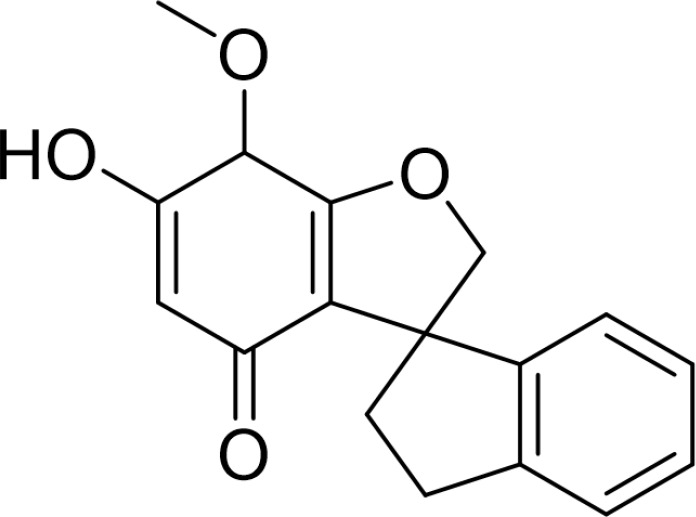
Tripdiolide	C_20_H_26_O_6_	362.42	Hydroxylated analog of TPL with poor aqueous solubility.	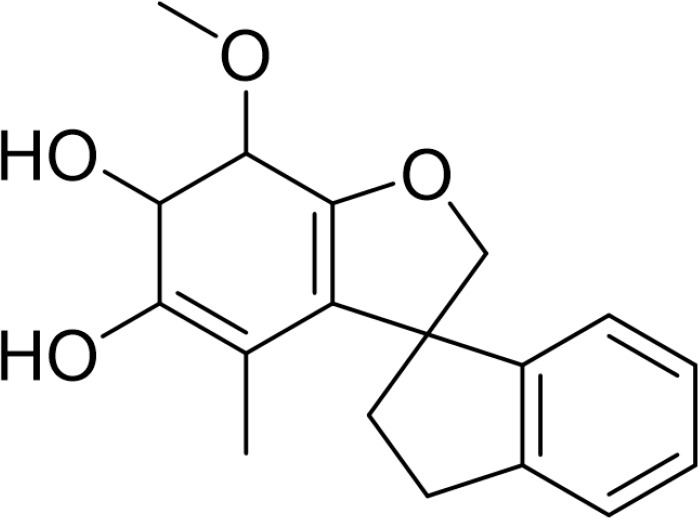
Triptonide	C_20_H_22_O_5_	342.39	Dehydrogenated TPL analog with conjugated double bonds and limited aqueous solubility.	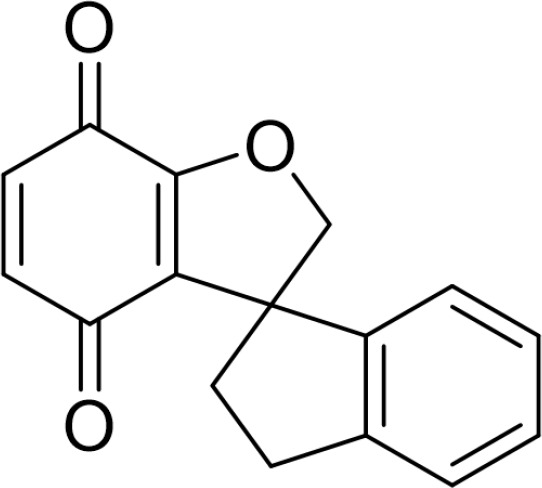
Celastrol	C_29_H_38_O_4_	450.62	Pentacyclic triterpenoid (oleanane-type) with a quinone-methide moiety; redox-active and poorly water-soluble.	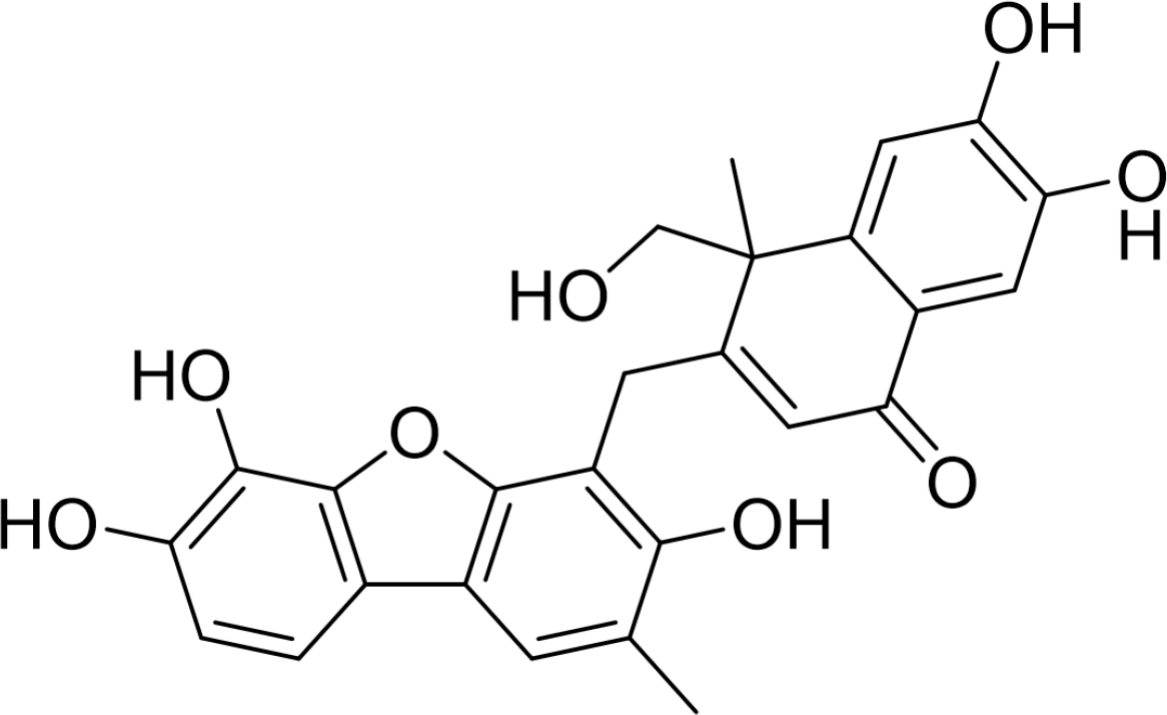
Wilforlide A	C_30_H_48_O_3_	456.71	Oleanane-type triterpenoid containing hydroxyl, carboxyl, and ketone groups; relatively lipophilic.	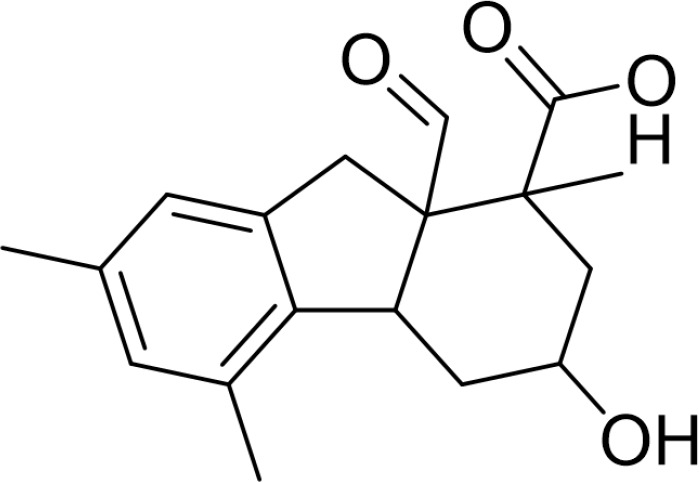
Wilforlide B	C_30_H_50_O_2_	442.72	Oleanane-type triterpenoid analog of wilforlide A; highly lipophilic with limited solubility in polar solvents.	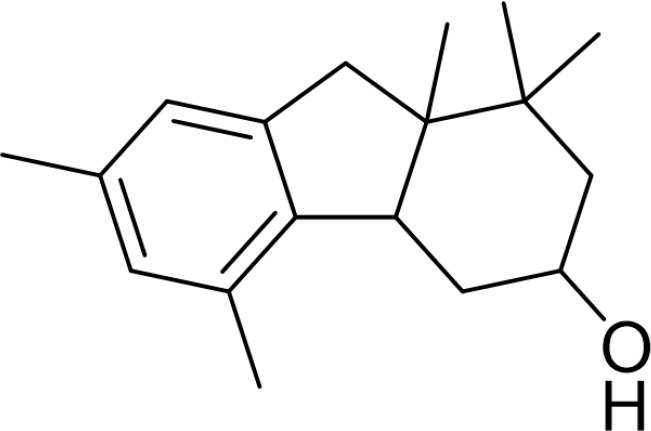
Tripterygium glycoside II	C_38_H_60_O_12_	724.88	Oleanane-type triterpenoid glycoside; glycosylation increases polarity compared with aglycone triterpenoids.	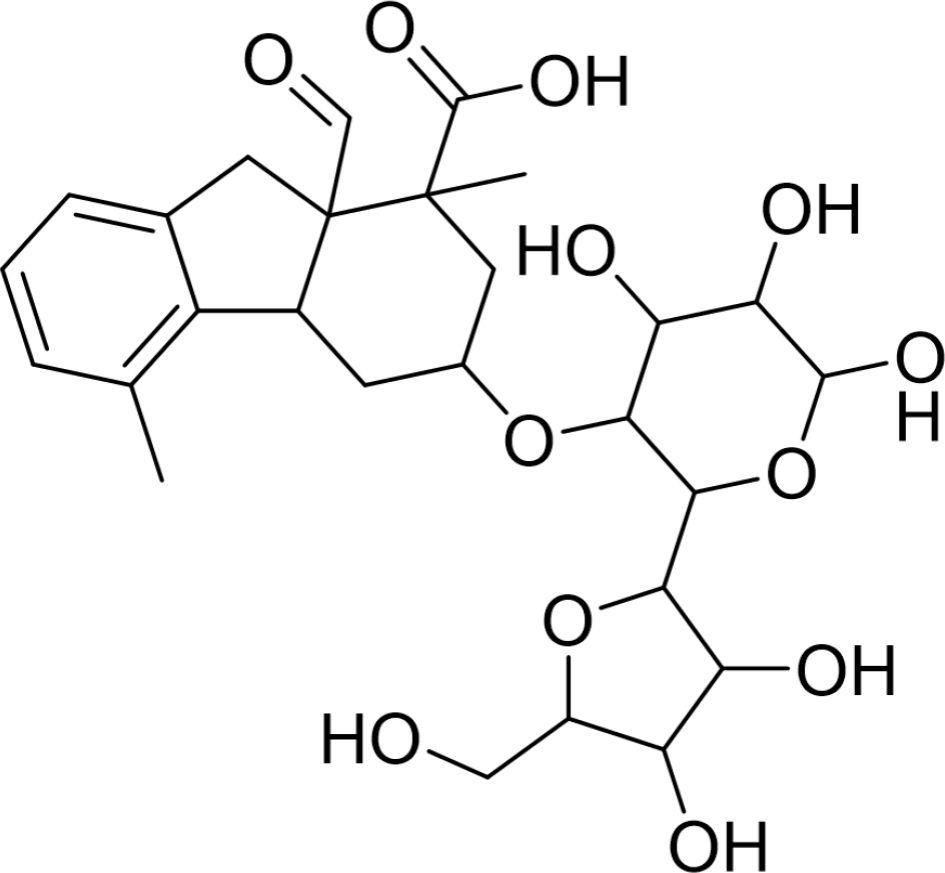
Wilfordine	C_41_H_49_NO_12_	747.83	Evonine-type alkaloid containing a β-carboline-related structure; nitrogen-containing and potentially salt-forming.	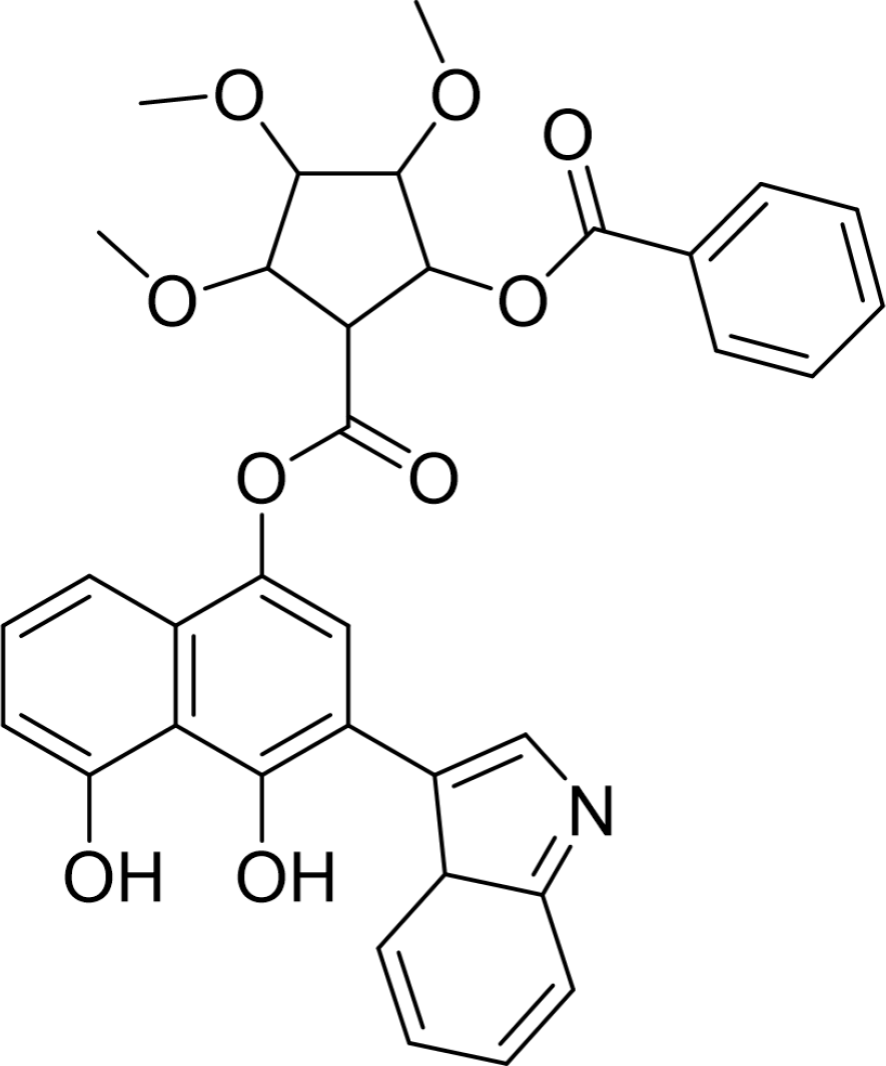
Wilforgine	C_42_H_51_NO_12_	761.86	Evonine-type alkaloid structurally related to wilfordine; lipophilic with limited aqueous solubility.	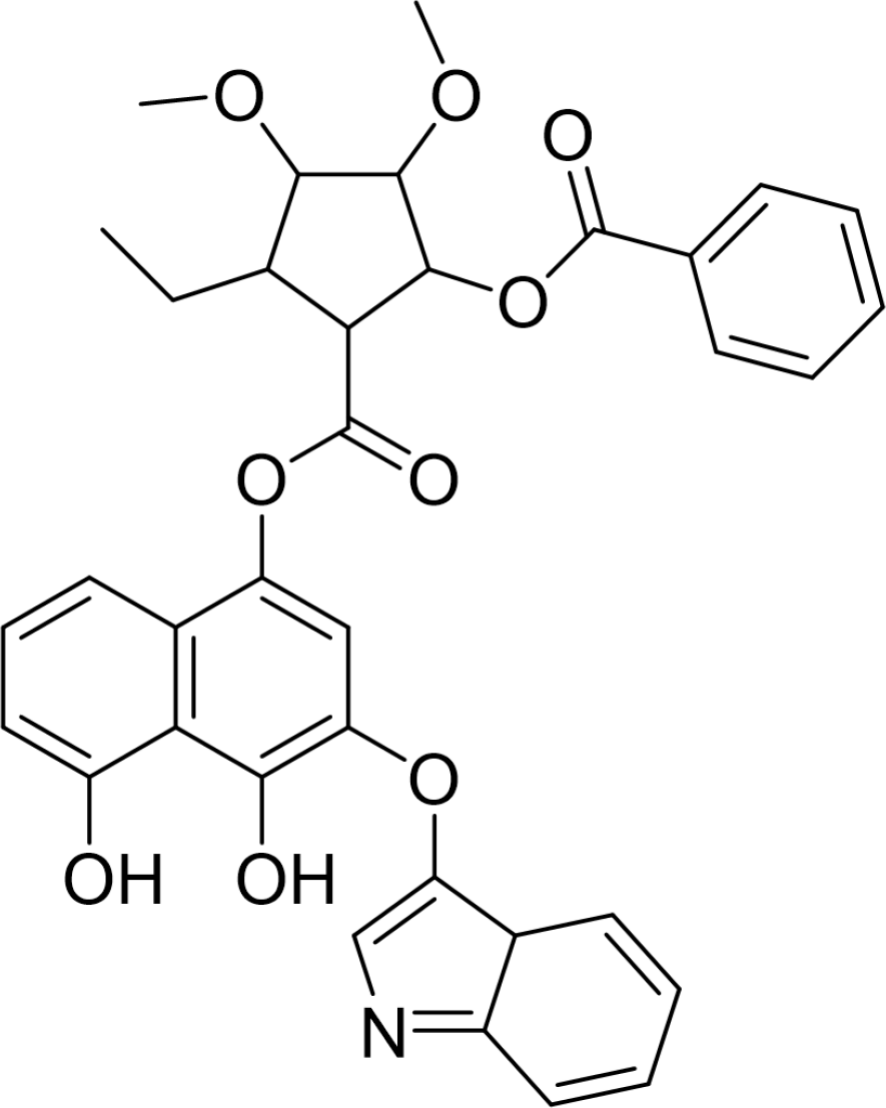
Tripterine	C_13_H_12_N_2_O	212.25	Dihydro-β-carboline alkaloid with a small nitrogen-containing scaffold.	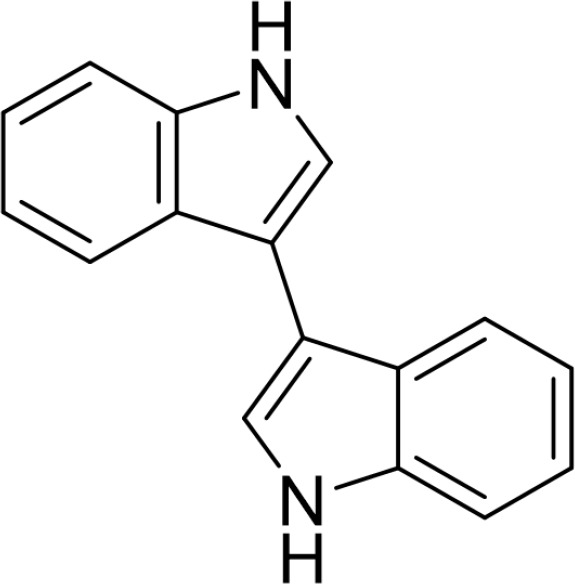
(5R)-5-Hydroxytriptolide (LLDT-8)	C_20_H_26_O_7_	378.42	Hydroxylated TPL derivative with a 5R-hydroxy substitution; designed to improve drug-development potential.	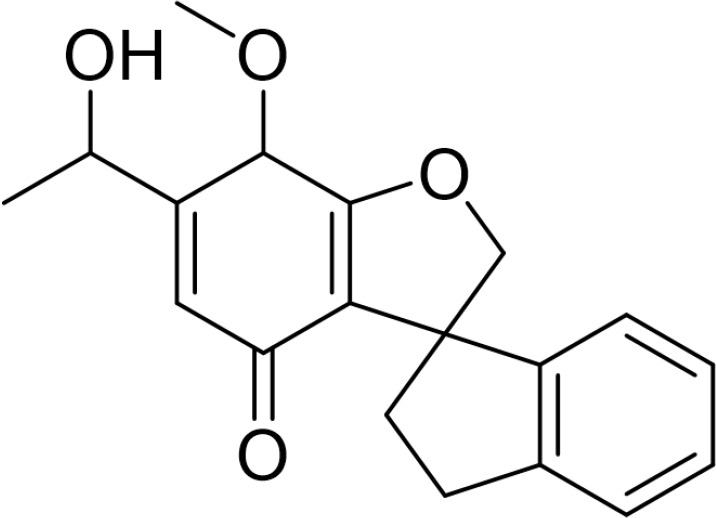
Mairin (XFC key active component)	C_16_H_12_O_7_	316.26	Benzopyran-containing polyphenolic compound with multiple hydroxyl groups; poorly water-soluble and soluble in organic solvents such as DMSO, ethanol, and acetone.	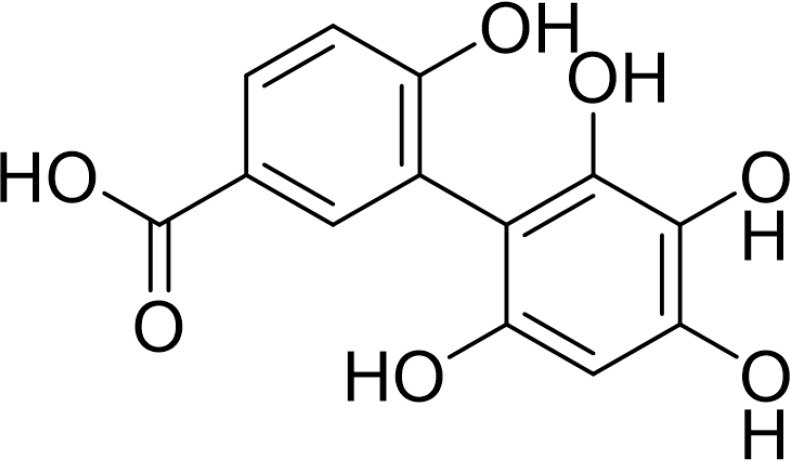

The molecular structures were drawn using ChemDraw 20.0 (PerkinElmer, USA). The molecular formula and weight, chemical classification, and structural information of each compound were cross-checked against PubChem. The corresponding PubChem CID numbers are provided to ensure structural traceability and chemical accuracy.

### 3.1 Diterpenoids

Diterpenoids represent the most well-characterized and pharmacologically potent class of monomers in TWHf. Among these, abietane-type diterpenoids are particularly important. Most of these compounds possess characteristic diterpenoid skeletons and are often substituted with epoxy, hydroxyl, or ketone groups. These groups are closely associated with their biological activities.

### 3.2 Triterpenoids

Triterpenoids in TWHf are mainly oleanane-type pentacyclic triterpenoids, which have a 30-carbon skeleton composed of six isoprene units. Compared with highly active diterpenoids [such as triptolide (TPL)], some triterpenoids may have lower cytotoxicity while retaining anti-inflammatory, immunomodulatory, hepatoprotective, and anti-proliferative activities. Their physicochemical properties depend largely on the number and position of hydroxyl, carboxyl, ketone, or acetyl groups on the pentacyclic backbone.

### 3.3 Alkaloids

Alkaloids from TWHf are nitrogen-containing heterocyclic compounds. They are primarily classified as dihydro-β-carboline alkaloids and evonine-type alkaloids. Compared with diterpenoids and triterpenoids, these monomers are relatively rare. However, they exhibit distinct pharmacological activities, such as anti-malarial and neuroprotective effects. Their basic nitrogen atoms confer alkaline properties, which enable salt formation with acids.

## 4 TWHf-derived monomers and preparations in RA: clinical evidence, molecular mechanisms, and translational considerations

TWHf-derived therapeutic agents in RA include individual bioactive monomers, optimized derivatives, total alkaloids, extracts, and total glycoside preparations. The following content is organized by pharmacological category and translational relevance to improve the logical flow of this section. First, core monomers and derivatives are summarized, with emphasis on their anti-inflammatory, immunomodulatory, anti-proliferative, anti-angiogenic, and bone-protective mechanisms. Next, extracts and total glycosides are discussed as multi-component preparations. Toxicity, biomarkers, and delivery strategies are presented separately to better distinguish mechanistic evidence from translational challenges.

### 4.1 Core monomers, derivatives, extracts, and total glycosides

#### 4.1.1 Celastrol

Celastrol is a key bioactive quinone-methide triterpenoid derived from TWHf. It has emerged as a promising therapeutic candidate for RA due to its potent immunomodulatory and anti-inflammatory properties (23). A previous study has demonstrated that celastrol suppresses adjuvant arthritis (AA) by inhibiting paw swelling, bone destruction, and pro-inflammatory cytokine expression (24). Building on this, recent studies have focused on structural optimization to enhance celastrol’s safety profile. For instance, semi-synthetic derivatives (such as COM5 and COM6) retain anti-arthritic activity while being significantly less toxic, partly due to modulation of calcium-associated unfolded protein response pathways (25).

Moreover, celastrol directly affects bone homeostasis by inhibiting osteoclast differentiation and function. In collagen-induced arthritis (CIA) models, it downregulates osteoclast-related genes and transcription factors (26). Further mechanistic insights reveal that celastrol targets multiple pathways (including the ALOX5-mediated TNF-NF-κB axis in macrophages), thereby reducing matrix metalloproteinase expression and alleviating bone erosion (27). Additionally, celastrol promotes apoptosis and cell cycle arrest in RA fibroblast-like synoviocytes (RA-FLS). This highlights its role in mitigating synovial hyperplasia (28).

Notably, celastrol also modulates autophagy via the PI3K/AKT/mTOR pathway, thereby enhancing autophagic activity in RA-FLS and subsequently improving arthritis symptoms *in vivo* (29). Celastrol has also been shown to suppress NLRP3 inflammasome activation by inhibiting the reactive oxygen species (ROS)-NF-κB axis, thereby reducing IL-1β and IL-18 secretion in both animal models and human macrophages (30). In summary, celastrol represents a multifaceted anti-RA agent, acting on osteoclastogenesis, synovial proliferation, autophagy, and inflammasome activity via interconnected signaling pathways.

#### 4.1.2 Total alkaloids of TWHf

In addition to individual bioactive compounds (such as celastrol), TWHf also exerts therapeutic efficacy via its total alkaloids (ATW). In CIA rat models, ATW treatment has been shown to significantly alleviate arthritis symptoms and joint damage by suppressing key inflammatory mediators (IL-6, IL-8, and TNF-α) and NF-κB; this suppression occurs both systemically and in synovial tissue (31). These findings highlight the potential of ATW as a multi-target botanical preparation for RA, further expanding the pharmacological profile of this traditional herb.

#### 4.1.3 Wilforine (WFR)

Other monomeric compounds from TWHf (e.g., WFR) also show notable anti-arthritic activity. It has been evidenced that WFR effectively ameliorates symptoms in CIA rats by suppressing the proliferation of synovial fibroblasts and the production of inflammatory cytokines (IL-6, IL-1β, and TNF-α) (32). Mechanistically, WFR exerts its effects by directly targeting Wnt11 and inhibiting the downstream Wnt/β-catenin signaling. These findings reveal a novel molecular axis for RA intervention and further expand the therapeutic options offered by TWHf-derived monomers.

#### 4.1.4 Tripterygium hypoglaucum (THH) extract

In addition to specific monomeric compounds, *Tripterygium* extracts also garner significant attention for their broad-spectrum efficacy in RA. Network pharmacology analyses of THH and TWHf have identified multiple active ingredients (e.g., TPL and celastrol) and key targets (e.g., PTGS2); these analyses also reveal that the synergistic action of these compounds is mediated primarily through inhibition of the TNF and NF-κB pathways (33, 34). Beyond direct anti-inflammatory effects, emerging evidence highlights a crucial gut-joint axis mechanism. A previous study has revealed that THH extract ameliorates adjuvant-induced arthritis (AIA) by modulating gut microbiota composition; it increases beneficial genera (e.g., *Bifidobacterium* and *Akkermansia*) while reducing harmful ones. This modulation subsequently suppresses the TLR4/MyD88/MAPK pathway (35). Moreover, metabolomic studies have indicated that THH extract rectifies metabolic disturbances in RA, notably by regulating the glutamine-glutamate cycle and targeting enzymes (e.g., GLUD2); this links immunomodulation to metabolic reprogramming (36). Concurrently, THH exerts systemic effects by inhibiting the NLRP3 inflammasome and remodeling bile acid profiles, underscoring the interconnection between gut microbiota, systemic inflammation, and joint pathology (37).

In summary, extracts from *Tripterygium* species exhibit multi-target, systemic therapeutic properties against RA. Their efficacy stems not only from direct modulation of inflammatory pathways (including NF-κB and TNF), but also from restoring gut microbiota homeostasis, regulating host metabolism, and suppressing inflammasome activation. This holistic mechanism highlights botanical extracts as valuable complex immunomodulators and supports their further development as integrative treatment strategies for RA.

#### 4.1.5 (5R)-5-Hydroxytriptolide (LLDT-8)

Emerging research underscores the therapeutic promise of novel derivatives from *Tripterygium*, such as LLDT-8. Bioinformatics analysis of RA synovial tissues has identified CD2 as a key gene in RA pathogenesis. Molecular docking then reveals that LLDT-8 binds to CD2 with high affinity; this binding inhibits CD2 expression and mitigates synovial cell inflammation (38). Furthermore, transcriptomic profiling of RA-FLS demonstrates that LLDT-8 induces broad gene expression changes, particularly downregulating immune and inflammatory pathways (e.g., cytokine-cytokine receptor interaction and TNF signaling). This indicates a systemic immunomodulatory effect (39). *In vivo* validation using a CIA model confirms that LLDT-8 not only alleviates arthritis severity but also modulates bone metabolism-related key pathways (including the OPG/RANK/RANKL axis); it also suppresses inflammatory mediators such as IL-1β, IL-6, and MMP-13 (40).

LLDT-8 exerts its therapeutic effects through multiple mechanisms. It directly targets critical immune molecules (such as CD2), reprograms the inflammatory transcriptome in synovial cells, and modulates bone remodeling and cytokine networks. Given these features and its improved safety profile over parent compounds, LLDT-8 is a promising candidate for targeted RA therapy.

### 4.2 Toxicity and safety concerns of TWHf-derived preparations

Although *Tripterygium* extracts and compounds show promising anti-RA efficacy, their clinical utility is significantly constrained by multi-organ toxicities. This necessitates a balanced evaluation of efficacy and safety. TGT exerts potent anti-inflammatory effects, partially by modulating glycerophospholipid and sphingolipid metabolism. However, it also induces hepatotoxicity through the same targets, such as ALOX5 and PTGS2 (41). In addition to hepatotoxicity, TGT administration is associated with marked male reproductive toxicity. Research indicates that this toxicity is mediated, at least in part, by inhibition of the NELL2-lumicrine system. This system regulates sperm maturation, and its disruption represents an off-target effect that undermines its therapeutic profile (42).

Overall, TGT reflects the dual nature of *Tripterygium*-based therapy: robust immunomodulation and systemic toxicity. Understanding TGT’s multi-target actions and its detrimental effects on reproductive and hepatic systems is crucial. Future development must focus on separating efficacy from toxicity, possibly via structural optimization or targeted delivery systems, enabling the safe use of its therapeutic potential.

### 4.3 Biomarkers and precision-response prediction

The clinical application of TGT in RA is constrained by significant variability in treatment response among patients. Several studies have focused on identifying predictive biomarkers to enable personalized therapy. A multi-omics analysis of transcriptomic profiles from patient peripheral blood cells has revealed several promising candidates. One study has identified six mRNA biomarkers: MX1, OASL, SPINK1, CRK, GRAPL, and RNF2. When these biomarkers are incorporated into a partial-least-squares (PLS) model, they significantly improve the prediction of therapeutic response compared to standard clinical parameters (43). In another study, through comprehensive analysis combining microRNA (miRNA) target prediction, co-expression networks, and gene-gene interactions, four circulating miRNA biomarkers have been found to predict TGT treatment response in RA. A robust support vector machine (SVM) prediction model is further constructed based on these miRNA levels. This model demonstrates excellent performance in both cross-validation and independent cohort validation for TGT treatment in RA (44). Moreover, another investigation has analyzed paired gene expression patterns, identified 17 co-expressed candidate gene pairs, and focused on the lnc-ENST00000602558/IGF1 axis (45). It’s confirmed that this axis mediates TGT efficacy, reduces TNF-α-induced IGF1 expression, and attenuates inflammation in MH7a cells.

Taken together, integrating transcriptomics and computational modeling has successfully identified multiple biomarkers that predict response to TGT, including mRNAs, miRNAs, and long non-coding RNA (lncRNA)-mediated axes. This progress underscores a pivotal shift towards precision medicine in RA management. These biomarkers can help identify potential responders early, maximizing efficacy and avoiding unnecessary exposure to a toxic drug, thereby optimizing the risk-benefit ratio of this potent botanical therapy.

### 4.4 Metabolic regulation by TGT

Beyond modulating immune and inflammatory pathways, TGT also plays a crucial role in rectifying RA-associated metabolic dysregulation, particularly in lipid metabolism. A previous study has revealed that in a CIA rat model, TGT effectively ameliorates arthritis symptoms and also corrects abnormal serum lipid levels; it notably reverses the elevation of pro-inflammatory ceramides and the depletion of lysophosphatidylcholines—both notably link to disease severity (46). Similarly, an integrated metabolomics and network analysis has identified that TGT treatment in CIA rats specifically modulates the glycerophospholipid metabolic pathway. It regulates key differential metabolites such as LysoP(18:0) and LysoPA(20:4) and involved hub genes (PLD1 and PLA2G4A) in this process (47).

Collectively, these studies demonstrate that TGT’s therapeutic efficacy also involves systemic metabolic reprogramming, specifically by normalizing dysregulated lipid metabolism. This reveals a new mechanism: lipid homeostasis may serve as a potential therapeutic target and a response biomarker in RA treatment.

### 4.5 TPL as a representative core monomer

#### 4.5.1 Inflammation

The specific bioactive component TPL exerts direct anti-inflammatory and immunomodulatory effects via well-defined molecular pathways. This drives its anti-RA effects in RA. Mechanistically, TPL inhibits the TREM1 pathway, downstream activation of JAK2/STAT3, and the production of pro-inflammatory cytokines (TNF-α, IL-1β, and IL-6) both *in vitro* and in CIA rats (48). This immunomodulation protects cartilage, as TPL treatment notably reduces TNF-α, IL-6, COX-2, and NF-κB expressions in the articular cartilage of arthritic rats (49). Furthermore, TPL also modulates the chemokine network. TPL effectively suppresses the overexpression of critical chemokines (e.g., MCP-1, MIP-1α, and RANTES) in the synovial tissue of AIA rats, thereby attenuating inflammatory cell recruitment (50). In addition to coding genes, recent research has uncovered a novel layer of TPL’s mechanism involving non-coding RNA networks. Specifically, TPL inhibits the proliferation and inflammatory response of RA-FLS by regulating the circ0003353/miR-31-5p/CDK1 competing endogenous RNA (ceRNA) axis, which subsequently manipulates the p21/Cyclin B cell cycle pathway. This axis also shows potential as a predictive biomarker for predicting TPL treatment response (51).

Overall, TPL acts as a multi-target therapeutic agent against RA inflammation, This highlights its central role within the broader pharmacological activity of TWHf-derived agents.

#### 4.5.2 Proliferation, invasion, and migration

TPL exerts a profound inhibitory effect on the pathogenic behaviors of RA-FLS, which are central to joint destruction. Primarily, TPL suppresses RA-FLS proliferation by blocking the JAK2/STAT3 pathway, thereby reducing the production of inflammatory cytokines (e.g., IL-6, IL-1β, and vascular endothelial growth factor (VEGF) induced by IL-6/sIL-6R complexes (52). Moreover, TPL notably reduces the migratory and invasive capacities of RA-FLS. This is partly due to inhibition of TNF-α-induced JNK phosphorylation within the MAPK pathway, which reduces F-actin polymerization and MMP-9 activity. These findings are validated in both *in vitro* models and a SCID mouse co-implantation assay (53). Furthermore, network pharmacology and molecular dynamics simulations reveal that TPL directly targets the RhoA/Rho-associated kinase (ROCK) signaling axis. By inhibiting RhoA/ROCK activation, TPL induces cytoskeletal remodeling. This remodeling is fundamental to suppressing RA-FLS motility and invasion (54). Importantly, TPL has been shown to exert anti-RA effects by targeting specific lncRNAs to modulate FLS behavior. For example, TPL suppresses FLS proliferation, invasion, and inflammation while promoting apoptosis by downregulating lncRNA RP11-83J16.1, which subsequently inactivates the URI1 and β-catenin pathways (55). Additionally, TPL downregulates ENST00000619282 in RA-FLS, shifting the balance toward pro-apoptotic and anti-inflammatory outcomes (56). Collectively, these studies demonstrate that TPL’s therapeutic efficacy is mediated, at least in part, by the coordinated inactivation of lncRNAs, which helps arrest pathogenic FLS activation in RA.

Taken together, TPL targets RA-FLS through multiple mechanisms. This broad action on synovial cells makes it effective against RA progression.

#### 4.5.3 Oxidative stress and autophagy

In addition to targeting proliferation and invasion, TPL also modulates critical cellular homeostatic processes in RA. In human synoviocyte MH7A cells, TPL significantly inhibits cell migration and invasion, and also maintains redox balance by enhancing antioxidant enzyme activities and reducing lipid peroxidation (57). Mechanistically, these effects are mediated by suppressing autophagy, which is mediated via the PI3K/AKT pathway activation. This highlights that TPL works against arthritis by linking oxidative stress and autophagic flux to synovial pathology.

#### 4.5.4 Macrophage polarization

Wan et al. have underscored a pivotal immunomodulatory mechanism of TPL: it acts not only on synoviocytes but also on innate immune cells (58). Specifically, TPL effectively reprograms macrophage polarization in RA by suppressing pro-inflammatory M1-secreted factors (e.g., IL-1β, TNF-α, and VEGF-A) and promoting anti-inflammatory M2 markers (IL-4 and IL-10). This restoration of M1/M2 homeostasis is achieved by inhibiting the NF-κB, PI3K/AKT, and p38 MAPK pathways, leading to ameliorated joint inflammation and restored immune imbalance.

#### 4.5.5 Th17 differentiation

Furthermore, TPL modulates adaptive immunity by targeting the Th17/Treg imbalance, a hallmark of RA pathogenesis (59). *In vitro* studies demonstrate that TPL inhibits pro-inflammatory Th17 cell differentiation in a dose-dependent manner. This inhibition is mediated by downregulated COX-2 expression and reduced PGE2 secretion by RA synovial fibroblasts (60). Notably, exogenous PGE2 reverses this inhibition, highlighting that the COX-2/PGE2/Th17 axis may serve as a key mechanistic target for TPL’s immunoregulatory action.

#### 4.5.6 Neutrophil regulation

TPL also modulates innate immune cells, particularly neutrophils, which are key drivers of inflammation in RA. In the AA murine model, TPL effectively alleviates disease by suppressing neutrophil recruitment, pro-inflammatory cytokine production, and key neutrophil effector functions [migration, neutrophil extracellular trap formation (NETosis), and autophagy], while promoting apoptosis (61). In a more recent study integrating network pharmacology and experimental validation, TPL alleviates CIA by reducing bone destruction and neutrophil infiltration. Crucially, TPL induces neutrophil apoptosis and inhibits NET formation and migration in a Hippo signaling pathway-dependent manner (62).

Together, these studies reveal that TPL exerts anti−arthritic effects largely by inducing apoptosis, suppressing migration and NETosis, and reducing inflammatory activation in abnormal neutrophils.

#### 4.5.7 Angiogenesis

Angiogenesis is a critical process sustaining synovial inflammation and pannus formation in RA. Kong et al. have revealed that TPL exerts anti-angiogenic effects in a CIA model (63). TPL treatment significantly reduces synovial blood vessel density and inhibits key angiogenesis steps, including endothelial cell adhesion, tube formation, and chemotactic migration. Mechanistically, TPL downregulates major angiogenic activators (VEGF, Ang-1/2, and Tie2) and suppresses the IL-1β-induced MAPK (ERK, p38, and JNK) pathway. This reflects its multi-target action on the vascular phase of RA.

#### 4.5.8 Omics-based mechanistic insights

Multi-omics approaches have revealed novel epigenetic and systemic mechanisms behind TPL’s therapeutic action in RA, going beyond traditional pathways. An integrated methylomic and transcriptomic analysis of RA patient peripheral blood mononuclear cells (PBMCs) revealed widespread dysregulation of RNA N6-methyladenosine (m6A) methylation. Fan et al. have highlighted IGF2BP3 as a key differentially methylated and expressed gene that is upregulated in RA synovium. Molecular docking and *in vitro* validation further confirm IGF2BP3 as a high-affinity target through which TPL exerts its regulatory effects (64). As revealed by a systemic proteomic analysis of serum exosomes from a CIA model, TPL treatment remarkably reverses disease-associated proteomic signatures. TPL suppresses pathways related to complement activation, IL-17 signaling, and cholesterol metabolism. Crucially, it downregulates the expression of specific exosomal and synovial proteins—LCN2 and MPO—both are implicated in neutrophil activity and oxidative stress (65).

Overall, these omics-driven studies identify IGF2BP3 as an epigenetic target and LCN2/MPO as systemic protein mediators. This expands its known mechanism from conventional inflammation to post-transcriptional regulation and cell-to-cell communication.

### 4.6 Delivery systems for toxicity reduction and efficacy enhancement

TPL has several limitations: poor bioavailability, systemic toxicity, and lack of targeting. To overcome these, advanced nanomedicine strategies have been developed to enhance its therapeutic potential. One approach uses folic acid-modified, self-assembled nanomicelles (FA-TP@VA NPs). This system targets inflammatory macrophages, improves drug accumulation in arthritic joints, and reduces systemic toxicity and hepatic oxidative stress compared with free TPL; it also suppresses synovitis and bone erosion in a mouse model (66). Another strategy focuses on localized intra-articular delivery. Researchers have fabricated TPL-loaded human serum albumin nanoparticles (TP@HSA NPs) and incorporated them into a thermosensitive hydrogel. This injectable depot system provides sustained local release of TPL within the joint,; it alleviates synovial inflammation and cartilage damage in CIA mice, with improved biosafety (67).

In summary, nanotechnology can deliver TPL via targeted systemic nanomicelles or localized hydrogel depots. This helps separate TPL’s anti-inflammatory effects from its off-target toxicity, making TPL safer and more effective for RA treatment.

**Figure 2:**
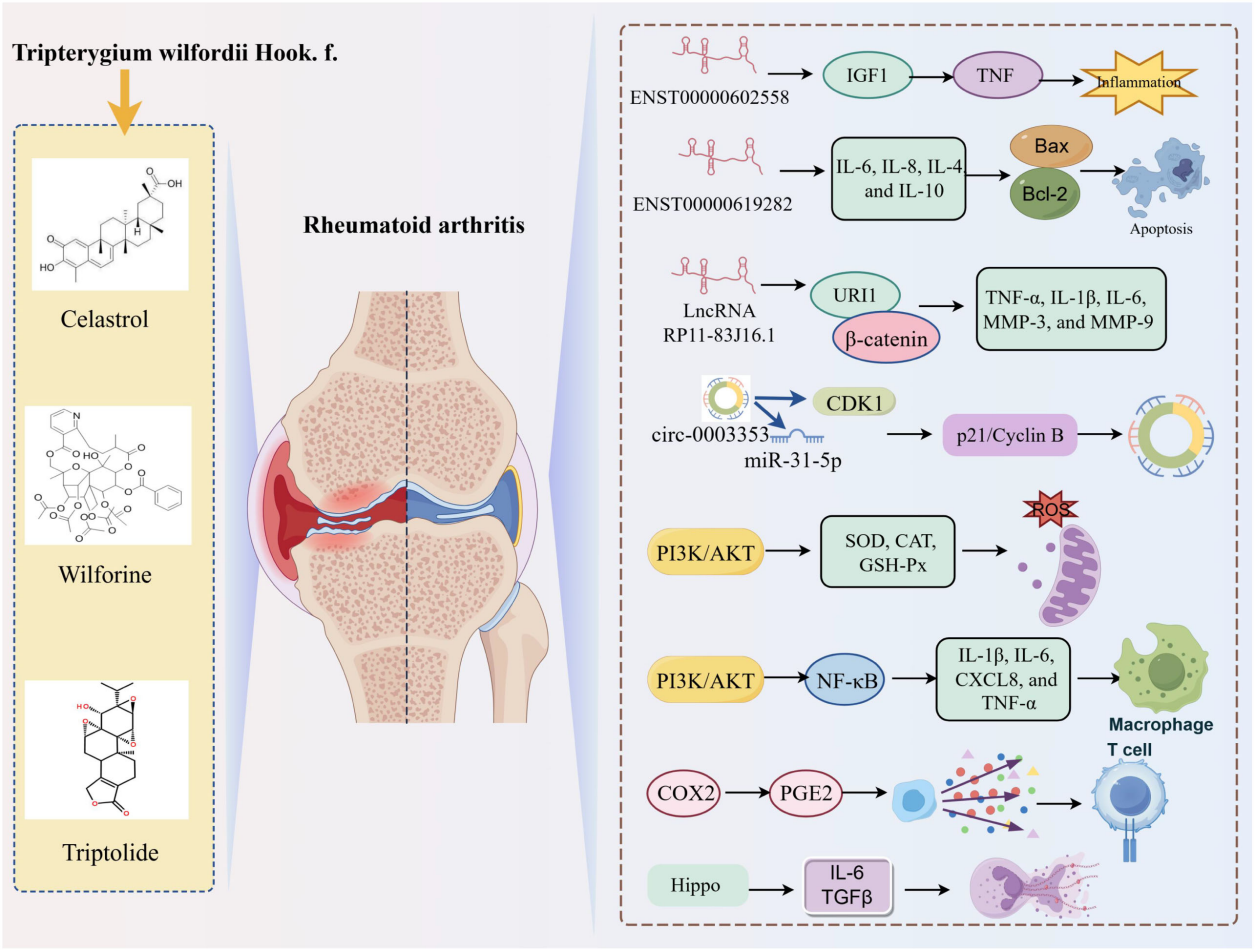
Multimodal mechanisms and signaling pathways targeted by TWHf bioactive monomers in RA pathogenesis. This schematic summarizes the major molecular targets and signaling pathways regulated by representative bioactive monomers from TWHf (including celastrol, WFR, and TPL) in RA. These compounds modulate multiple RA-related pathological processes, including synovial inflammation, FLS activation, apoptosis, oxidative stress, macrophage activation, T-cell responses, neutrophil activity, and angiogenesis. The right panel highlights key molecular axes involved in these effects, such as ENST00000602558/IGF1/TNF, ENST00000619282-mediated inflammatory and apoptotic regulation, lncRNA RP11-83J16.1/URI1/β-catenin, circ0003353/miR-31-5p/CDK1/p21-Cyclin B, PI3K/AKT-mediated oxidative stress regulation, PI3K/AKT/NF-κB-mediated macrophage inflammation, COX2/PGE2-mediated T-cell modulation, and Hippo-related neutrophil regulation. RA, rheumatoid arthritis; TWHf, *Tripterygium wilfordii* Hook. f.; TNF, tumor necrosis factor; IL, interleukin; MMP, matrix metalloproteinase; ROS, reactive oxygen species; SOD, superoxide dismutase; CAT, catalase; GSH-Px, glutathione peroxidase; NF-κB, nuclear factor kappa B; COX2, cyclooxygenase-2; PGE2, prostaglandin E2.

TWHf and its key bioactive constituents (including celastrol, TPL, and total alkaloids) work against RA through multiple pathways. They provide potent anti-inflammatory, immunomodulatory, and joint-protective effects (**Table 2**; **Figure 2**).

**Table 2:** Molecular targets and mechanisms of key bioactive monomers derived from TWHf in RA: a systematic summary of preclinical evidence.

TWHf	Models	Targets	Mechanisms
Celastrol	AA rats	IL-1β/TNF-α	Suppresses IL-1β and TNF-α expression to reduce AA inflammation.
	AIA rats	COM5/COM6	CEL derivatives COM5/COM6 mitigate RA via Ca²^+^-associated UPR with reduced toxicity.
	CIA mice and RAW264.7 cell line	RANKL	Inhibits RANKL-induced osteoclastogenesis and bone erosion.
	Macrophages	ALOX5/NF-κB/MMP	Celastrol mitigates RA bone erosion by inhibiting macrophage ALOX5/NF-κB/MMP signaling.
	RA-FLS	Bax/Bcl-2/caspase9	Celastrol induces DNA damage, G2/M arrest, and apoptosis in RA-FLS.
	CIA mouse	PI3K/AKT/mTOR	Celastrol ameliorates RA by inhibiting PI3K/AKT/mTOR to upregulate autophagy.
	CFA and THP-1 cells	ROS/NF-κB/NLRP3	Celastrol alleviates RA by inhibiting the ROS/NF-κB/NLRP3 inflammasome axis.
ATW	CIA rats	IL-6, IL-8, and TNF-α	Reduces inflammatory cytokine production in CIA rats.
WFR	RA-FLS	Wnt11/β-catenin	WFR alleviates RA by inhibiting FLS activation via the Wnt11/β-catenin pathway.
THH extract	AIA rats	TLR4/MyD88/MAPK	THH extract alleviates AIA by modulating gut microbiota and inhibiting TLR4/MyD88/MAPK.
	AA rats	GLUD2	Regulates glutamine-glutamate metabolism through GLUD2.
	CFA	NLRP3	THH alleviates RA via bile acid-mediated gut-joint axis and inhibiting NLRP3 inflammasome.
LLDT-8	Synovial tissues of RA patients	CD2	LLDT-8 ameliorates RA by targeting CD2 to inhibit synovial inflammation.
	RA-PBMCs	miR-146a	TWHf alleviates RA by downregulating circulating miR-146a, correlating with disease activity.
	RA-FLS	/	LLDT-8 modulates RA-FLS transcriptome, particularly immune-related pathways and lncRNA networks.
	CIA rats	MMP-13 and OPG/RANKL	LLDT-8 ameliorates CIA by inhibiting MMP-13 and modulating OPG/RANKL signaling.
TGT	CIA rats	IL-1β	Corrects lipid metabolic imbalance and reduces IL-1β-mediated inflammation.
	CIA rats	PLD1, LPCAT4, AGPAT1, and PLA2G4A	TGT ameliorates CIA by modulating glycerophospholipid metabolism and lipid-related hub genes.
TPL	CIA rats	TREM1-DAP12/JAK2-STAT3	TPL ameliorates RA by inhibiting TREM1-DAP12/JAK2-STAT3 signaling and cytokine production.
	AA rats	TNF-α, IL-6, NF-κB, and COX-2	TPL ameliorates CIA by inhibiting TNF-α, IL-6, NF-κB, and COX-2 in cartilage.
	AA rats	MCP-1, MIP-1α	TPL ameliorates AA by inhibiting MCP-1, MIP-1α, and RANTES expression.
	RA patients and RA-FLS	circ0003353/miR-31-5p/CDK1	TPL inhibits RA-FLS growth via circ0003353/miR-31-5p/CDK1 ceRNA axis.
	RA-FLS	IL-6/sIL-6R and JAK2/STAT3	TPL inhibits IL-6/sIL-6R-induced FLS proliferation via JAK2/STAT3 pathway.
	RA-FLS	JNK/MMP-9	TPL inhibits RA-FLS migration/invasion and CIA via suppressing JNK/MMP-9 signaling.
	RA-FLS	RhoA/ROCK	TPL inhibits RA-FLS motility via RhoA/ROCK-mediated cytoskeleton remodeling.
	MH7A	AKT	TPL inhibits MH7A cell mobility and oxidative stress by suppressing autophagy via AKT.
	Macrophages	NF-κB/PI3K/p38	TPL restores macrophage M1/M2 balance by inhibiting NF-κB/PI3K/p38 pathways in RA.
	Th17 cell	COX-2/PGE2	TPT inhibits RASF COX-2/PGE2 and subsequent Th17 cell differentiation.
	Neutrophils	NETosis	Inhibits neutrophil recruitment, NETosis, and inflammatory activation.
	Neutrophils	Hippo	Promotes neutrophil apoptosis and suppresses NET formation via Hippo signaling.
	CIA rats	VEGF and MAPK	Inhibits VEGF/MAPK-mediated synovial angiogenesis.
	RA-FLS	LncRNA RP11-83J16.1/URI1/β-catenin	Suppresses RA-FLS activation through the RP11-83J16.1/URI1/β-catenin axis.
	RA-FLS	ENST00000619282	Downregulates ENST00000619282 to promote apoptosis and reduce inflammation.

### 4.7 TWHf as a positive control drug and toxicity-reduction reference

Although Tripterygium wilfordii extracts demonstrate potent efficacy against RA, their clinical utility is significantly constrained by dose-limiting toxicities, particularly hepatotoxicity. A mechanistic study has revealed that TW-induced liver injury involves the LXRα-ERS-SREBP-1c pathway activation, leading to abnormal hepatic lipid synthesis. Notably, a compound formula containing TW, Qingluotongbi (QLT), mitigates this hepatotoxicity by inhibiting this specific pathway, thereby separating efficacy from toxicity (68). Given this toxicity profile, purified Tripterygium preparations are often used as a benchmark in pharmacological research. For instance, in a study of Qingluoyin (QLY) granules, TGT served as the positive control drug. The study demonstrates that QLY granules exert comparable therapeutic efficacy to TG tablets in an AA model, effectively reducing joint inflammation and suppressing the CXCL12/CXCR4-NF-κB pathway (69).

Tripterygium derivatives are highly effective, which makes them a gold-standard comparator in RA studies. Additionally, they also remind us that we need new formulations or combinations to keep the efficacy while improving safety.

### 4.8 Reduced toxicity and increased efficacy when combined with other TCM

In TCM, TWHf is often combined with other herbs to reduce its hepatotoxicity and multi-organ damage without compromising efficacy. One strategy involves combining Tripterygium wilfordii with Spatholobus suberectus Dunn (JXT). This compatibility not only enhances the anti-inflammatory and analgesic effects of LGT but also significantly reduces its hepatotoxicity. The protective mechanism is linked to the downregulated VEGFA/Flt-1 signaling pathway and reduced content of the toxic component TPL (70). Similarly, another effective pairing involves combining TGT with extracts from Salvia miltiorrhiza Bunge (71). Specifically, in a CIA model, the hydrophilic salvianolic acids (SA) and lipophilic tanshinones (Tan) from Salvia enhance the anti-arthritic efficacy of TGT while alleviating TWP-induced damage to the liver, kidney, and testis.

These studies collectively validate the TCM principle of herbal compatibility, demonstrating that scientifically designed combinations can preserve Tripterygium’s efficacy while reducing its toxicity.

### 4.9 Clinical evidence from RCTs

RCTs are prospective studies in which participants are randomly assigned to an experimental or control group. This methodological gold standard is predicated on the principle of randomization, effectively mitigating selection bias and ensuring the baseline comparability of key prognostic variables between the groups. By establishing a robust causal framework, RCTs generate high-level evidence essential for evaluating the efficacy and safety of therapeutic interventions in RA.

#### 4.9.1 Combination with biologics

Emerging clinical evidence supports the promising role of TWHf in combination with biologics for RA. In a multi-center RCT, in MTX-inadequate responders, adalimumab (ADA) plus TWHf demonstrates comparable efficacy and safety to the standard ADA plus MTX regimen over 24 weeks, suggesting TWHf as a viable alternative for MTX-intolerant patients (72). This finding indicated that TWHf serves as an effective alternative to MTX in biologic combination therapy; it also possesses unique properties that enhance the clinical response to TNF inhibitors, potentially offering a more effective option for active RA.

#### 4.9.2 Combination with DMARDs

The long-term extension study of the RCTs provides critical evidence on the sustained efficacy of TWHf. Over a 2-year period, TWHf alone is as effective as MTX in controlling disease activity and slowing radiographic progression in DMARD-naïve active RA patients (73). Importantly, the TWHf + MTX combination also shows comparable long-term efficacy and safety. Consistently, another study has demonstrated that at 24 weeks, TWHf monotherapy achieves a significantly higher ACR50 response rate (55.1%) compared to MTX monotherapy (46.4%), demonstrating its non-inferiority (74). Notably, the TWHf + MTX combination therapy is superior to MTX alone, with a markedly higher ACR50 response (76.8%). These findings confirm that TWHf provides long-term benefits and can serve as an alternative or complementary therapy to MTX for RA treatment.

An RCT in U.S. centers first shows that TWHf is effective in patients with active RA (75). Compared to sulfasalazine, TWHf extract demonstrates significantly superior efficacy at 24 weeks. Analyses show that among TWHf-treated patients, 65.0% achieve an ACR20 response, compared with only 32.8% in the sulfasalazine group. TWHf also led to rapid reductions in serum IL-6 levels. Importantly, despite a high dropout rate, the frequency of adverse events was comparable between groups, suggesting an acceptable risk-benefit profile for TWHf.

## 5 XFC (a compound Tripterygium preparation) in RA: clinical application and mechanistic insights

XFC, a TCM compound preparation, was developed by Professor Liu Jian from the First Affiliated Hospital of Anhui University of Chinese Medicine based on the Xin’an medicine theory of “strengthening the root and cultivating yuan, treating bi syndrome from the spleen”. As a promising therapeutic agent for RA, XFC has been included in the Guidelines for Integrated Disease-Syndrome Diagnosis and Treatment of Rheumatoid Arthritis formulated by the Chinese Association of Traditional Chinese Medicine in 2025 (76; (21). A previous meta-analysis investigating XFC’s efficacy in RA treatment demonstrates that compared with leflunomide (LEF), XFC could alleviate joint swelling and pain, reduce morning stiffness duration, and decrease the levels of ESR, CRP, and anti-CCP antibody (77). These findings confirm that XFC exerts therapeutic effects on RA, as reflected by remarkable improvement in joint symptoms and inflammatory indicators of RA patients. Additionally, RCTs, cohort studies, and data-mining studies have shown that XFC is effective in RA. Furthermore, systematic and in-depth studies on the molecular mechanisms underlying XFC’s therapeutic effects on RA have been conducted.

Unlike isolated TWHf-derived monomers, XFC contains multiple herbs that work together across different biological pathways. Therefore, its therapeutic value should not be interpreted simply as the sum of individual signaling pathways. Instead, XFC may exert integrated anti-RA effects by coordinating multiple mechanisms, including inflammation control, immune homeostasis, synovial protection, oxidative-stress regulation, vascular remodeling, coagulation balance, and extra-articular protection. More importantly, the compatibility of its herbal components may make XFC more effective and less toxic than single TWHf monomers or extracts. This is a core advantage of the formula. Accordingly, this section was reorganized according to integrated pathological modules rather than isolated pathway entries.

### 5.1 Integrated regulation of inflammation and immune dysregulation

#### 5.1.1 Inflammatory signaling

RA is a chronic autoimmune disorder driven by synovial inflammation and the associated signaling pathways. Network pharmacology and *in vivo* validation identified several bioactive components in XFC, including mairin and TPL. These components target AKT1 and TNF to suppress synovial hyperplasia and inflammation in RA (78). In another study, XFC alleviates RA inflammatory symptoms by downregulating the TLR pathway, reducing levels of inflammatory markers (e.g., CRP, VEGF) and pro-inflammatory cytokines (e.g., TNF-α, IL-6, and IFN-γ), and inhibiting the expression of TLR4/MyD88/NF-κB cascade components (79). Furthermore, Cao et al. have confirmed that XFC also attenuates cardiac injury in AA rats by suppressing the TLR4/MAPK/NF-κB pathway, decreasing myocardial inflammatory cytokines (TNF-α, IL-17, and IL-6), and inhibiting cardiomyocyte apoptosis (80). Taken together, these findings highlight that XFC reduces inflammation in both joints and cardiac tissues by interfering with TLR signaling.

#### 5.1.2 Neutrophil NETosis and epigenetic regulation

NETosis drives RA pathogenesis by exacerbating synovial inflammation, oxidative stress, and autoimmune responses through extracellular chromatin release. A recent study has shown that neutrophil dysregulation, particularly through NETosis, plays a pivotal role in RA pathogenesis. A clinical and mechanistic investigation demonstrates that XFC safely ameliorates RA immune and inflammatory markers (81). Crucially, its therapeutic effect is mediated by targeting the ALKBH5-m6A-LINC00968 axis, which epigenetically regulates neutrophil hyperactivation. XFC inhibits ALKBH5 activity, which increases m6A methylation of LINC00968. This in turn suppresses NET formation, oxidative stress (via the NADP+/NADPH pathway), and pro-inflammatory cytokine release. This work identifies a novel pathway by which XFC attenuates the neutrophil-NET-inflammation cascade, providing an epigenetic strategy for RA treatment.

### 5.2 Regulation of synovial cell fate and tissue remodeling

#### 5.2.1 Cuproptosis

Cuproptosis, a novel copper-dependent form of regulated cell death, contributes to chondrocyte damage in RA. Wang et al. have revealed that XFC attenuates RA-associated joint injury by targeting the METTL3/miR-221/222-3p/ATP7A axis to suppress chondrocyte cuproptosis (82). These changes reduce intracellular copper accumulation, ROS, mitochondrial dysfunction, and inflammatory cytokine release, along with key cuproptosis drivers (FDX1, LIAS, and DLAT). Consequently, XFC protects chondrocytes from cuproptosis-induced damage.

#### 5.2.2 Proliferation, apoptosis, and autophagy

Dysregulated proliferation and impaired apoptosis of RA-FLS are pivotal in driving synovial hyperplasia and joint destruction in RA. Recently, a mechanistic study has revealed that XFC targets this pathogenic process through multiple mechanisms. One study demonstrates that in TNF-α-stimulated RA-FLS, XFC upregulates the circular RNA circ-CBLB. This leads to cell cycle arrest (increased S and G2 phases) and enhanced apoptosis, and shifts the cytokine balance toward an anti-inflammatory profile, with elevated IL-4 and IL-10 and reduced IL-6 and TNF-α. Together, these effects suppress FLS hyperproliferation (83). Moreover, another investigation elucidates an RNA epigenetic pathway mediated by the demethylase FTO. FTO reduces m6A modification on the lncRNA ENST00000619282, which stabilizes the lncRNA and promotes its YTHDF1−dependent expression. This lncRNA inhibits PUF60 and activates the NF−κB pathway. The result is that FLS escape from apoptosis (19). XFC blocks NF−κB signaling through two mechanisms: downregulating FTO (which increases m6A methylation of ENST00000619282) and direct binding of its active components to NF−κB p65. This restores FLS apoptosis and inhibits proliferation.

Overall, XFC counteracts RA-FLS pathogenic activation by modulating circular RNA (circRNA) and m6A-modified lncRNAs and inhibiting NF-κB. This restores apoptotic sensitivity and suppresses proliferative synovitis.

Impaired autophagy in RA leads to synovial hyperplasia and sustained inflammation by dysregulating cellular homeostasis. A study on AA rats demonstrates that XFC effectively enhances autophagy by upregulating key markers (e.g., LC3-II and Beclin1) while inhibiting the PI3K/AKT/mTOR pathway (84). XFC treatment also rebalances inflammatory cytokines, reducing IL-1β and TNF-α while increasing IL-4 and IL-10. These findings indicate that XFC exerts anti-arthritic effects partially through restoring autophagy and modulating inflammatory responses, suggesting that autophagy is a therapeutic target in RA.

#### 5.2.3 Pyroptosis

Cellular pyroptosis is a pro-inflammatory form of programmed cell death, which exacerbates synovitis and systemic damage in RA by releasing IL-1β and IL-18. Wang et al. have shown that XFC-containing serum suppresses LPS-induced pyroptosis in RA-FLS by downregulating the NLRP3/caspase-1/GSDMD pathway, leading to reduced secretion of IL-1β and IL-18 (85). Additionally, another study has revealed that XFC also mitigates myocardial injury in AIA rats by modulating the lncRNA GAS5/miR-21/TLR4 axis (86). This regulation subsequently inhibits myocardial pyroptosis, as evidenced by decreased NLRP3, caspase-1, and GSDMD expressions and reduced serum levels of pro-inflammatory cytokines. This effect is enhanced by GAS5 overexpression or the pyroptosis inhibitor NSA. XFC inhibits pyroptosis in both joints and hearts of RA models. It targets the canonical NLRP3 inflammasome pathway in synovium and the GAS5/miR-21/TLR4 axis in myocardium.

#### 5.2.4 Angiogenesis and synovial remodeling

Pathological angiogenesis is a hallmark of RA, promoting synovial hyperplasia and joint destruction. Liu et al. have reported that XFC mitigates RA-FLS-induced angiogenesis in human umbilical vein endothelial cells (HUVECs) by targeting the lncRNA HOTAIR/miR-126-3p/PI3K/AKT axis (87). XFC suppresses HUVEC proliferation, migration, tube formation, and endothelial marker (CD34/CD105) expression, downregulates VEGF and bFGF, and inhibits the PI3K/AKT signaling. These findings indicate that XFC attenuates synovial angiogenesis through epigenetic and post-transcriptional regulation, highlighting its potential as an anti-angiogenic agent in RA therapy.

### 5.3 Systemic complications and extra-articular protection

#### 5.3.1 Hypercoagulability and coagulation homeostasis

Hypercoagulability represents a critical pathological feature in RA, which is closely linked to chronic inflammation. This contributes to both joint damage and cardiovascular complications. Emerging evidence highlights the therapeutic potential of XFC in ameliorating this prothrombotic state. One clinical study demonstrates that XFC significantly improves TCM blood stasis syndrome and reduces coagulation markers (e.g., D-dimer, fibrinogen). These changes are associated with suppression of the Act1/NF-κB pathway and downregulated pro-inflammatory and pro-coagulant mediators (e.g., IL-6 and IL-17) and platelet-activating factor (88). Another investigation, integrating clinical data mining, animal models, and *in vitro* co-culture systems, further reveals that XFC exerts dual anti-inflammatory and anticoagulant effects, at least in part, by inhibiting the PI3K/AKT pathway in RA (89). This action leads to the reduction of key coagulation-related factors (such as tissue factor). In addition, active components of XFC show strong binding affinity to HIF1A and PTGS2. Collectively, these findings suggest that XFC mitigates RA-associated hypercoagulability by targeting both inflammation and coagulation, supporting its use in patients with a prothrombotic phenotype.

#### 5.3.2 Oxidative stress and extra-articular injury

Oxidative stress is a pivotal driver in RA pathogenesis, exacerbating synovial inflammation, joint destruction, and systemic extra-articular complications. Emerging research underscores the potent anti-oxidative properties of XFC in mitigating these pathological processes. One study has demonstrated that XFC alleviates RA-associated lung injury by regulating the PPARγ/HMGCS2 pathway (90). Specifically, XFC-containing serum suppresses TGF-β1-induced oxidative stress and fibrosis in lung fibroblasts; it reduces ROS, NOX4, and collagen deposition while enhancing PPARγ expression. Furthermore, another study has revealed that XFC upregulates the lncRNA LINC00638 in RA-FLS, thereby activating the Nrf2/HO-1 antioxidant pathway. This leads to decreased levels of IL-6, IL-17, ROS, and reactive nitrogen species (RNS), alongside increased expression of SOD2 and HO-1 (91). Rescue experiments confirm that silencing LINC00638 attenuates XFC’s protective effects, further highlighting the functional role of this lncRNA in mediating XFC’s anti-oxidative action.

#### 5.3.3 RA with cardiac involvement

RA often involves cardiac complications, contributing to increased cardiovascular risk through persistent inflammation and immune dysregulation. It has been evidenced that XFC exhibits cardioprotective effects in RA models via multi-target modulation of inflammatory and fibrotic pathways. In AA rats, XFC improves cardiac function indicators (e.g., LVSP, CI), attenuates myocardial structural damage, and suppresses the TLR4/MyD88/NF-κB cascade, leading to reduced expression of downstream pro-inflammatory mediators (such as TNF-α) (84). Furthermore, XFC modulates the TGF-β1/Smads pathway by downregulating Smad2/4 and upregulating the inhibitory Smad7, thereby decreasing immune complex deposition and myocardial fibrosis (92). At the cellular level, XFC-containing serum enhances viability and reduces apoptosis in LPS-injured H9C2 cardiomyocytes. This effect is mediated partly by upregulation of miRNA-21 and inhibition of the TLR4/p-p38/p-p65 axis, along with reduced pro-inflammatory cytokine release (TNF-α, IL-6, IL-17) (93).

**Figure 3:**
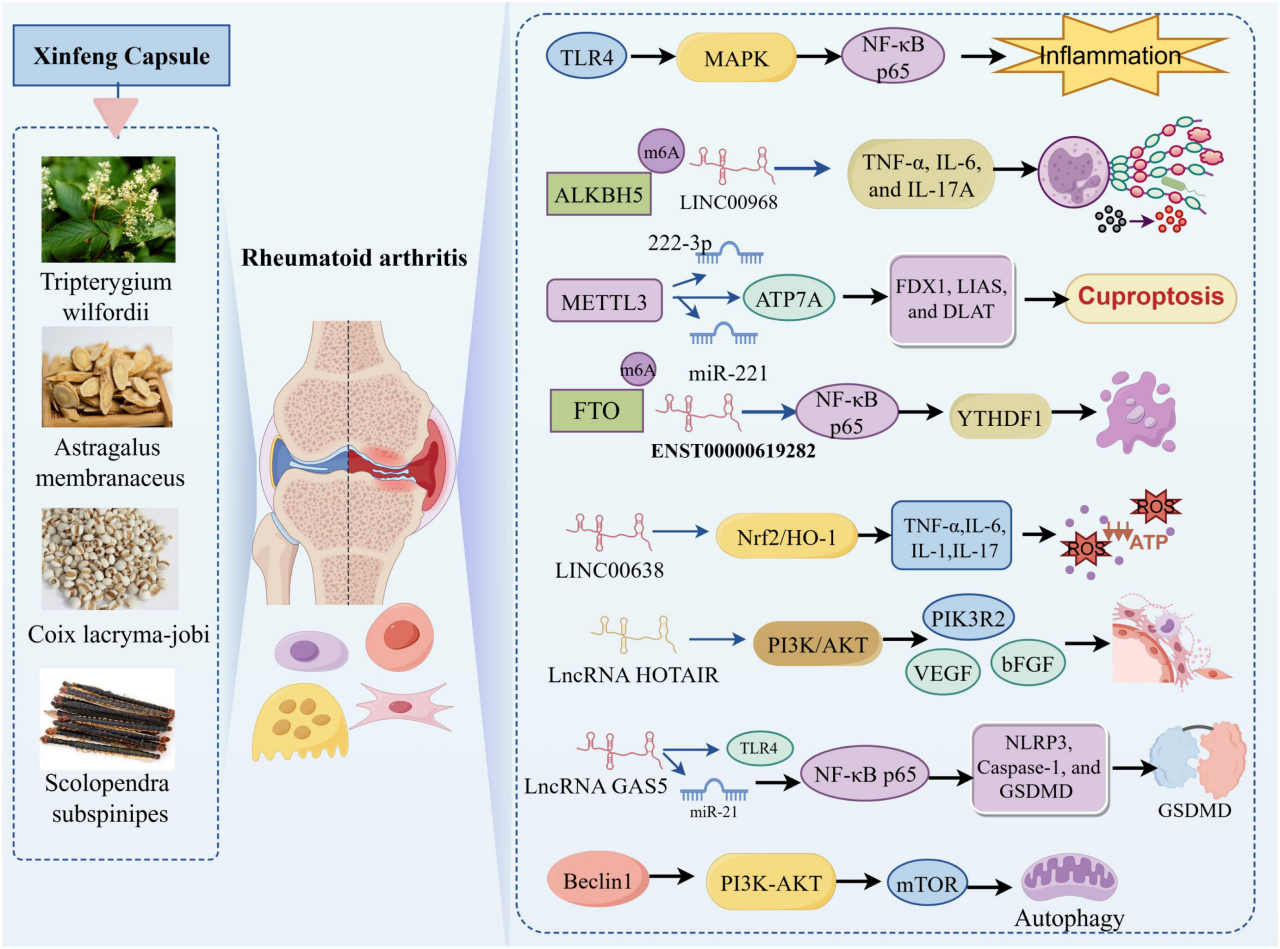
Integrated molecular network and systemic protective effects of XFC in RA. This schematic summarizes the composition and major molecular mechanisms of XFC in RA. XFC is composed of *Tripterygium wilfordii*, *Astragalus membranaceus*, *Coix lacryma-jobi*, and *Scolopendra subspinipes*. The right panel illustrates the multi-target regulatory effects of XFC on RA-related pathological processes, including inflammatory signaling, neutrophil NETosis, cuproptosis, apoptosis, oxidative stress, angiogenesis, pyroptosis, and autophagy. Representative pathways include TLR4/MAPK/NF-κB-mediated inflammation, ALKBH5/m6A/LINC00968-mediated NETosis, METTL3/miR-221/ATP7A-mediated cuproptosis, FTO/ENST00000619282/YTHDF1/NF-κB-mediated apoptosis regulation, LINC00638/Nrf2/HO-1-mediated oxidative-stress control, lncRNA HOTAIR/PI3K/AKT-mediated angiogenesis, lncRNA GAS5/miR-21/TLR4/NF-κB-mediated pyroptosis, and Beclin1/PI3K-AKT/mTOR-mediated autophagy. Together, these mechanisms highlight the multi-component and multi-target regulatory features of XFC in RA treatment. XFC, Xinfeng Capsule; RA, rheumatoid arthritis; TLR4, Toll-like receptor 4; MAPK, mitogen-activated protein kinase; NF-κB, nuclear factor kappa B; NETosis, neutrophil extracellular trap formation; m6A, N6-methyladenosine; TNF-α, tumor necrosis factor-α; IL, interleukin; ROS, reactive oxygen species; VEGF, vascular endothelial growth factor; bFGF, basic fibroblast growth factor; GSDMD, gasdermin D; mTOR, mammalian target of rapamycin.

Taken together, these findings indicate that XFC exerts protective effects on RA by attenuating inflammatory activation, inhibiting fibrotic signaling, and enhancing cardiomyocyte survival. This multi-mechanistic action makes it a promising agent against RA-associated cardiovascular pathology (**Table 3**; **Figure 3**).

**Table 3:** Summary of molecular mechanisms underlying the therapeutic effects of XFC on RA.

Molecular phenotype	TCM	Model	Targets	Mechanisms
Inflammation	XFC	AA rats	TLR	XFC alleviates RA inflammation via modulating the TLR signaling pathway.
Inflammation	XFC	AA rats	TLR4/MAPK/NF-κB	XFC attenuates inflammation and cardiac injury in AA rats by inhibiting the TLR4/MAPK/NF-κB signaling pathway.
NETosis	XFC	RA patients and neutrophil	ALKBH5/LINC00968	XFC attenuates RA inflammation by targeting the ALKBH5-m6A-LINC00968 axis to inhibit neutrophil NETosis.
Cuproptosis	XFC	AA rats and chondrocyte	METTL3/miR-221-3p/ATP7A	XFC alleviates RA joint damage by inhibiting chondrocyte cuproptosis via METTL3/miR-221-3p/ATP7A axis.
Hypercoagulable state	XFC	RA patients	Act1/NF-κB	XFC ameliorates RA blood stasis by inhibiting the Act1/NF-κB signaling pathway.
Hypercoagulable state	XFC	AA rats and RA-PBMCs co-cultured with VECs	PI3K/AKT	XFC exerts anti-inflammatory and anticoagulant effects in RA by inhibiting the PI3K/AKT pathway.
Proliferation and apoptosis	XFC	RA-FLS	circ-CBLB	XFC induces RA-FLS apoptosis and anti-inflammatory response via upregulating circ-CBLB expression.
Proliferation and apoptosis	XFC	RA-PBMCs co-cultured with RA-FLS	FTO/ENST00000619282/YTHDF1/NF-κB	XFC inhibits FLS apoptosis escape by modulating the FTO-m6A-ENST00000619282/YTHDF1/NF-κB axis.
Oxidative stress	XFC	AA rats	PPARγ/HMGCS2	XFC mitigates RA-associated lung injury via the PPARγ/HMGCS2 pathway.
Oxidative stress	XFC	RA patients, AA rats and RA-FLS	LINC00638/Nrf2/HO-1	XFC alleviates RA via upregulating LINC00638 to activate the Nrf2/HO-1 antioxidant pathway.
Angiogenesis	XFC	RA-FLS co-cultured HUVEC	lncRNA HOTAIR/PI3K/AKT	XFC inhibits RA synovial angiogenesis via suppressing the lncRNA HOTAIR/PI3K/AKT pathway.
Pyroptosis	XFC	RA-FLS	NLRP3/GSDMD	XFC mitigates RA joint inflammation by inhibiting NLRP3/GSDMD-mediated FLS pyroptosis.
Pyroptosis	XFC	AA rats	LncRNA GAS5/miR-21/TLR4	XFC alleviates myocardial injury in AA rats by modulating the GAS5/miR-21/TLR4 axis and inhibiting pyroptosis.
Autophagy	XFC	AA rats	PI3K/AKT/mTOR	XFC enhances synovial autophagy in AA rats via modulating the PI3K/AKT/mTOR pathway.
RA Cardiac involvement	XFC	AA rats	TLR4/NF-κB	XFC protects cardiac function in AA rats by inhibiting the TLR4/NF-κB signaling pathway.
RA Cardiac involvement	XFC	AA rats	Smad7/NF-κB/TGF-β1/Smads	XFC improves cardiac function in AA rats by upregulating Smad7 to inhibit NF-κB and TGF-β1/Smads pathways.
RA Cardiac involvement	XFC	AA rats and H9C2 cells	miR-21/TLR4/p38/NF-kB	XFC serum protects H9C2 cells via upregulating miR-21 and inhibiting TLR4/p38/NF-kB pathway.

### 5.4 Compatibility-based advantages of XFC: efficacy enhancement and toxicity reduction

The above evidence suggests that the therapeutic value of XFC lies in its compound compatibility, enabling multi-pathway regulation in RA. XFC is a formulation composed of Astragali Radix et Rhizoma, Coicis Semen, Tripterygii Radix et Rhizoma, and Scolopendra. The formula has both immune-regulating and anti-inflammatory effects, and also helps resolve dampness and dredge collaterals. Compared with isolated TWHf monomers, this compound may target a wider range of RA processes with lower toxicity risk. Mechanistically, the compatibility of XFC allows it to act on multiple targets, including inflammatory cytokines, synovial fibroblast activation, neutrophil NETosis, regulated cell death, oxidative stress, angiogenesis, and coagulation imbalance. This multi-target action achieves network-level regulation rather than single-pathway inhibition.

Importantly, this compatibility may explain the improved efficacy and reduced toxicity of XFC. By combining TWHf with supportive and modulatory herbs, XFC may preserve the potent anti-inflammatory and immunomodulatory effects of TWHf while improving tolerability through herb interactions. This distinguishes XFC from single monomers (such as TPL or celastrol), which often have narrow therapeutic windows and dose-dependent toxicity. Nevertheless, the precise synergistic, additive, or detoxifying interactions among XFC components remain unclear and should be further validated using pharmacokinetic analysis, chemical fingerprinting, systems pharmacology, and well-designed clinical safety studies.

### 5.5 Clinical evidence from RCTs

Evidence from multiple RCTs elucidates the multifaceted therapeutic potential of XFC in RA. A multi-center RCT has demonstrated that XFC is as effective as LEF in improving core disease activity measures such as the Disease Activity Score in 28 joints (DAS28) and the American College of Rheumatology (ACR) response criteria. However, it reveals a distinct advantage in alleviating depressive symptoms, as reflected by greater reduction in the Self-Rating Depression Scale (SDS) scores (21). Subsequent *post-hoc* analyses of this trial further identify that XFC has an advantage over LEF in improving coagulation and platelet parameters (FBG, PLT, and PCT), which are linked to RA disease activity and quality of life (94). Moreover, XFC’s benefits appear to extend beyond articular and hematological domains. As indicated by another RCT specifically targeting RA patients with impaired pulmonary function, compared to Tripterygium glycosides, XFC not only relieves joint symptoms but also improves lung function [Forced expiratory flow at 50% of forced vital capacity (FEF50) and Diffusing capacity of the lung for carbon monoxide (DLco)] and overall quality of life (77). Collectively, these findings position XFC as a promising therapeutic agent capable of improving joint inflammation, coagulation abnormalities, and extra-articular pulmonary manifestations in RA.

### 5.6 Cohort study

In contrast to the tightly controlled framework of RCTs, cohort studies provide important real-world evidence for evaluating XFC’s long-term effectiveness and safety in heterogeneous RA populations. Large cohort studies provide longitudinal data on safety, comorbidities, and healthcare use in large patient populations. This information cannot be obtained from RCTs. It helps guide long-term clinical management and health economic evaluations.

**Figure 4:**
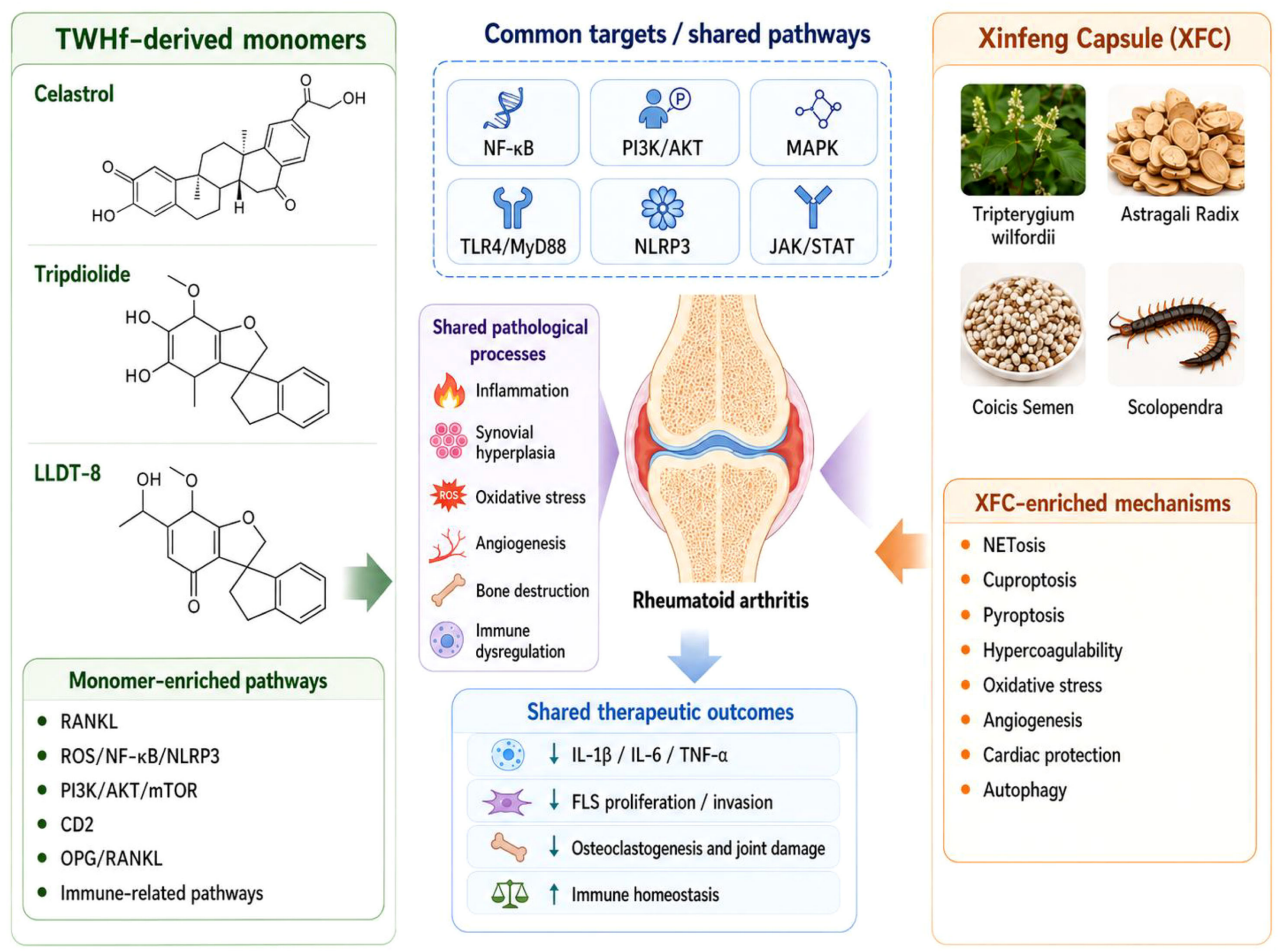
Common and distinct molecular mechanisms of TWHf-derived monomers and XFC in RA. This schematic summarizes the shared and distinct mechanisms of TWHf-derived monomers and XFC in RA. The left panel shows representative TWHf-derived monomers, including celastrol, tripdiolide, and LLDT-8, together with their relatively enriched pathways. The central panel summarizes common targets and shared pathological processes regulated by both TWHf-derived monomers and XFC, including NF-κB, PI3K/AKT, MAPK, TLR4/MyD88, NLRP3 inflammasome, JAK/STAT, inflammation, synovial hyperplasia, oxidative stress, angiogenesis, bone destruction, and immune dysregulation. The right panel highlights XFC as a multi-component preparation with XFC-enriched mechanisms, including NETosis, cuproptosis, pyroptosis, hypercoagulability, oxidative stress, angiogenesis, cardiac protection, and autophagy. Compared with isolated monomers, XFC may provide compatibility-based advantages through multi-component regulation, broader systemic protection, potential efficacy enhancement, and toxicity reduction.

Real-world cohort studies can complement RCTs by providing long-term data on the clinical impact of therapies (such as XFC) in RA. Firstly, a large retrospective cohort employing propensity score matching demonstrated that XFC markedly reduces the long-term risk of hospital readmission. The benefit is more pronounced after 12 months of treatment (22). Furthermore, subsequent analyses confirmed and extended this finding. Another study has indicated that XFC alone and XFC-containing formulas improved both lab parameters and patient-reported outcomes, reflecting benefits on inflammation and quality of life (95). Notably, use of these TCM formulas was associated with a lower risk of a composite endpoint that included readmission, Sjögren’s syndrome, surgical intervention, and all-cause mortality. A clear dose-response relationship is observed: more treatment is linked to greater risk reduction (96). Importantly, XFC may also benefit certain challenging patient subgroups. A specific cohort focusing on RA patients with comorbid hyperlipidemia (RA-HL) confirms that XFC-containing herbal regimens were linked to a lower risk of readmission (97). In summary, this series of well-designed cohort studies consistently shows that XFC improves long-term clinical outcomes (including reduced hospitalizations and complications) across broad and specific RA populations. These findings suggest that XFC is effective in real-world RA care. To integrate the mechanistic findings, we generated a schematic diagram comparing the shared targets and distinct regulatory features of TWHf-derived monomers and XFC in RA (**Figure 4**). In addition, **Table 4** summarizes the differences between monomer-based and compound-formula-based interventions, including their efficacy, mechanisms, toxicity profiles, advantages, limitations, and translational implications.

**Table 4:** Comparison of TWHf-derived monomers and XFC in RA.

Item	TWHf-derived monomers	XFC
Representative form	Single monomers/derivatives	Multi-component compound preparation
Representative examples	TPL, celastrol, LLDT-8, WFR	TWHf, Astragali Radix, Coicis Semen, Scolopendra
Efficacy	Potent anti-inflammatory and immunomodulatory effects	Broader clinical improvement in joint and systemic manifestations
Mechanisms	Relatively defined pathways (NF-κB, PI3K/AKT, JAK/STAT, ROS/NLRP3, RANKL)	Integrated regulation of inflammation, NETosis, cuproptosis, pyroptosis, oxidative stress, angiogenesis, and coagulation
Toxicity	More prominent toxicity concerns, especially for TPL-related agents	Potentially better tolerability through compatibility, but further safety validation is needed
Advantages	Clearer targets and stronger suitability for mechanistic drug development	Multi-component synergy, broader systemic protection, and potential efficacy enhancement/toxicity reduction
Limitations	Narrow therapeutic window and insufficient clinical validation	Complex active basis and incomplete mechanistic deconvolution
Translational significance	Lead compounds for precision or targeted therapy	Clinically oriented integrative therapeutic strategy

### 5.7 Evidence quality and safety assessment of clinical studies

To improve the transparency of clinical evidence, we summarized the risk of bias, certainty of evidence, and safety profiles of the RCTs discussed in this review. Risk of bias was considered across key domains, including random sequence generation, allocation concealment, blinding, incomplete outcome data, selective reporting, and other potential biases. The certainty of evidence was assessed using the GRADE framework, based on risk of bias, inconsistency, indirectness, imprecision, and publication bias. Evidence for improvements in disease activity, joint symptoms, and inflammatory markers was generally moderate. Evidence for extra-articular outcomes, long-term safety, and subgroup-specific benefits was low to moderate, due to limited sample sizes, insufficient blinding, short follow-up, and incomplete adverse event reporting.

Safety data from clinical studies were also summarized. Reported adverse reactions associated with TWHf-derived preparations mainly included gastrointestinal discomfort, liver function abnormalities, hematological changes, reproductive or menstrual-related adverse effects, renal function abnormalities, and infection-related risks. XFC was generally reported to have a relatively favorable safety profile compared with single TWHf-derived preparations or highly active monomers, though long-term safety data are still lacking. In clinical practice, liver and renal function, blood routine examination, inflammatory markers, and reproductive safety should be regularly monitored. Dose reduction, temporary interruption, or discontinuation should be considered when clinically significant adverse events occur. TWHf-related preparations should be used cautiously or avoided in pregnant or lactating women, patients preparing for pregnancy, and individuals with severe liver, kidney, blood, or active infectious conditions.

## 6 Challenges and future perspectives

Despite progress in understanding TWHf and XFC for RA, several challenges remain that limit their clinical use and optimization.

For TWHf-derived monomers, their clinical utility is significantly hampered by dose-limiting systemic toxicities, including hepatotoxicity, reproductive toxicity, and nephrotoxicity. Moreover, inherent physicochemical properties (such as poor water solubility and low oral bioavailability) further impede effective drug delivery. Personalized treatment is still limited by a lack of validated biomarkers to predict response or toxicity, so dosing remains one-size-fits-all.

Regarding the compound preparation XFC, XFC appears effective and safer than single monomers, but the current evidence still has limitations. There is a scarcity of large-scale, multi-center RCTs with rigorous methodology and long-term follow-up. Furthermore, the mechanisms behind XFC’s herbal compatibility, whether synergistic, additive, or detoxifying, need more scientific validation. The precise molecular targets and the contributions of its multi-component system to the observed multi-target effects require further elucidation using advanced analytical technologies.

A common overarching challenge is the lack of stratified clinical research. Most studies treat RA as a homogeneous entity, with few investigations into how responses differ across subtypes (e.g., seropositive vs. seronegative, early vs. established disease) or patient endotypes defined by specific molecular signatures.

From a clinical and translational perspective, several practical issues should be addressed before the broader application of TWHf-related preparations and XFC. Dosage and treatment duration should follow approved instructions, local guidelines, or regimens used in well-designed clinical studies, and empirical dose escalation should be avoided. During treatment, liver and renal function, complete blood count, inflammatory markers, and reproductive safety should be regularly monitored; these preparations should be used cautiously or avoided in pregnant or lactating women, patients preparing for pregnancy, and individuals with severe liver, kidney, blood, or active infectious conditions. International translation also remains challenging because TWHf- and XFC-based therapies differ from conventional Western small-molecule drugs in several ways: they are multi-component, have different regulatory status, and face issues with extract standardization, batch consistency, and quality control. In the next 3-5 years, Future research should prioritize several areas: multi-center RCTs, standardized chemical fingerprinting and active-component quantification, biomarker-guided patient stratification, toxicity-reducing delivery systems, and integrative omics- or AI-assisted studies to clarify how XFC works and support its precision use in RA.

Another challenge is whether current findings apply to different patient populations. Most available clinical evidence for TWHf-derived preparations and XFC has been generated in Chinese populations, whereas ethnic pharmacogenomic differences in drug metabolism, immune responses, comorbidities, and concomitant medication use may affect both efficacy and toxicity. Therefore, whether the clinical benefits and safety profiles observed in existing studies can be directly extrapolated to non-Chinese or multi-ethnic populations requires further validation. In addition, the regulatory status of TWHf-based preparations and compound TCM formulations differs substantially outside China, which may limit their clinical application. Standardization remains a major challenge for future multi-center trials. Variations in plant origin, cultivation, harvesting, extraction, quality control, and batch consistency could affect reproducibility and comparability. Therefore, future international studies should emphasize standardized manufacturing processes, chemical fingerprinting, active-compound quantification, and harmonized safety-monitoring protocols to improve the global translational potential of TWHf- and XFC-based therapies.

An interdisciplinary approach is needed to address these challenges:

1. Monomer optimization and delivery: Advanced formulation strategies, such as nanotechnology-based delivery systems (e.g., targeted nanomicelles, hydrogels), prodrug design, and structural modification, should be prioritized to enhance bioavailability, achieve tissue-specific targeting, and dissociate efficacy from toxicity.
2. Deciphering compound formula mechanisms: Systems biology approaches (including integrative network pharmacology, metabolomics, and gut microbiome analysis) can help explain the synergistic principles of XFC and provide a molecular view of TCM compatibility.
3. Deepening mechanistic insights: cutting-edge tools (e.g., single-cell RNA sequencing and spatial transcriptomics) are needed to map the precise effects of TWHf/XFC on specific immune cell subsets and stromal cells within the synovial microenvironment, revealing cell-type-specific targets and pathways.
4. Advancing precision medicine: Prospective biomarker discovery programs should integrate multi-omics data (genomics, transcriptomics, proteomics) from well-characterized patient cohorts. This is essential for identifying signatures that predict response and toxicity, enabling biomarker-guided personalized therapy.
5. Robust clinical validation: There is an urgent need for rigorously designed, large-scale, Phase III/IV RCTs and longitudinal real-world evidence studies to confirm the efficacy, safety, and cost-effectiveness of XFC. Research should also optimize its integration into combination regimens with conventional DMARDs or biologics.
6. Leveraging artificial intelligence (AI): AI and machine learning algorithms can help analyze complex high-dimensional datasets (clinical, omics, imaging), predict drug-target interactions, optimize formulation design, and identify patient subgroups that are most likely to benefit. This could accelerate drug development and personalization.

## 7 Conclusions

In summary, TWHf-derived monomers and XFC represent two complementary therapeutic paradigms for RA. Individual monomers (such as TPL and celastrol) provide relatively defined molecular targets and potent anti-inflammatory or immunomodulatory effects, whereas XFC reflects the compound-formula strategy of multi-component, multi-target, and network-level regulation. This distinction is important because XFC may work not only through pathway modulation but also through herb interactions that boost efficacy and lower toxicity.

Nevertheless, several challenges still limit the broader use of TWHf and XFC therapies: dose-limiting toxicity of certain monomers, insufficient standardization of botanical preparations, limited high-quality multicenter trials, incomplete safety-monitoring frameworks, and a lack of validated biomarkers for patient stratification. Therefore, future research should shift focus from descriptive pathway analysis to several priorities: standardized quality control, rigorous clinical evidence generation, systematic adverse-event monitoring, pharmacokinetic and pharmacodynamic characterization, and biomarker-guided precision application. Addressing these issues will help move TWHf and XFC therapies from promising but experimental interventions to safer, more evidence-based options for RA.

## Glossary

AA: Adjuvant arthritis
CIA: Collagen-induced arthritis
RA: Rheumatoid arthritis
AIA: Adjuvant-induced arthritis
CFA: Complete Freund’s adjuvant
TWHf: Tripterygium wilfordii Hook. f.
XFC: Xinfeng Capsule
TGT: Tripterygium Glycosides Tablets
TPL: Triptolide
DMARD(s): Disease-modifying antirheumatic drug(s)
NSAID(s): Non-steroidal anti-inflammatory drug(s)
MTX: Methotrexate
LEF: Leflunomide
QLY: Qingluoyin
QLT: Qingluotongbi
ATW: The total alkaloids of Tripterygium wilfordii Hook f.
RA-FLS: Rheumatoid arthritis fibroblast-like synoviocytes
PBMC(s): Peripheral blood mononuclear cell(s)
HUVEC(s): Human umbilical vein endothelial cell(s)
NF-κB: Nuclear factor kappa B
PI3K/AKT: Phosphoinositide 3-kinase/Protein kinase B
mTOR: Mammalian target of rapamycin
MAPK: Mitogen-activated protein kinase
JAK/STAT: Janus kinase/Signal transducer and activator of transcription
TLR: Toll-like receptor
MyD88: Myeloid differentiation primary response 88
NLRP3: NLR family pyrin domain containing 3
VEGF: Vascular endothelial growth factor
ROS: Reactive oxygen species
RNS: Reactive nitrogen species
m6A: N6-methyladenosine
lncRNA: Long non-coding RNA
circRNA: Circular RNA
miRNA: MicroRNA
ceRNA: Competing endogenous RNA
RCT: Randomized controlled trial
TCM: Traditional Chinese Medicine
ACR: American College of Rheumatology
DAS28: Disease Activity Score in 28 joints
SDS: Self-Rating Depression Scale
FEF50: Forced expiratory flow at 50% of forced vital capacity
DLco: Diffusing capacity of the lung for carbon monoxide
NETosis: Neutrophil extracellular trap formation.
